# Unraveling COVID-19: a large-scale characterization of 4.5 million COVID-19 cases using CHARYBDIS

**DOI:** 10.21203/rs.3.rs-279400/v1

**Published:** 2021-03-01

**Authors:** Daniel Prieto-Alhambra, Kristin Kostka, Talita Duarte-Salles, Albert Prats-Uribe, Anthony Sena, Andrea Pistillo, Sara Khalid, Lana Lai, Asieh Golozar, Thamir M Alshammari, Dalia Dawoud, Fredrik Nyberg, Adam Wilcox, Alan Andryc, Andrew Williams, Anna Ostropolets, Carlos Areia, Chi Young Jung, Christopher Harle, Christian Reich, Clair Blacketer, Daniel Morales, David A. Dorr, Edward Burn, Elena Roel, Eng Hooi Tan, Evan Minty, Frank DeFalco, Gabriel de Maeztu, Gigi Lipori, Heba Alghoul, Hong Zhu, Jason Thomas, Jiang Bian, Jimyung Park, Jordi Martínez Roldán, Jose Posada, Juan M Banda, Juan P Horcajada, Julianna Kohler, Karishma Shah, Karthik Natarajan, Kristine Lynch, Li Liu, Lisa Schilling, Martina Recalde, Matthew Spotnitz, Mengchun Gong, Michael Matheny, Neus Valveny, Nicole Weiskopf, Nigam Shah, Osaid Alser, Paula Casajust, Rae Woong Park, Robert Schuff, Sarah Seager, Scott DuVall, Seng Chan You, Seokyoung Song, Sergio Fernández-Bertolín, Stephen Fortin, Tanja Magoc, Thomas Falconer, Vignesh Subbian, Vojtech Huser, Waheed-Ul-Rahman Ahmed, William Carter, Yin Guan, Yankuic Galvan, Xing He, Peter Rijnbeek, George Hripcsak, Patrick Ryan, Marc Suchard

**Affiliations:** Centre for Statistics in Medicine (CSM), Nuffield Department of Orthopaedics, Rheumatology and Musculoskeletal Sciences (NDROMS), University of Oxford, UK; Real World Solutions, IQVIA, Cambridge, MA, USA; Fundació Institut Universitari per a la recerca a l’Atenció Primària de Salut Jordi Gol i Gurina (IDIAPJGol), Barcelona, Spain; Centre for Statistics in Medicine, University of Oxford; Janssen R&D, Titusville NJ, USA, 2) Department of Medical Informatics, Erasmus University Medical Center, Rotterdam, The Netherlands; Fundació Institut Universitari per a la recerca a l’Atenció Primària de Salut Jordi Gol i Gurina (IDIAPJGol), Barcelona, Spain; Centre for Statistics in Medicine, NDORMS, University of Oxford, UK; Division of Cancer Sciences, School of Medical Sciences, University of Manchester, UK; Regeneron Pharmaceuticals, NY USA, Department of Epidemiology, Johns Hopkins Bloomberg School of Public Health, MD USA; Medication Safety Research Chair, King Saud University, Riyadh, Saudi Arabia; National Institute for Health and Care Excellence, London, UK; School of Public Health and Community Medicine, Institute of Medicine, Sahlgrenska Academy, University of Gothenburg, Gothenburg, Sweden; Department of Biomedical Informatics and Medical Education, University of Washington, Seattle, WA, USA, 2) UW Medicine, Seattle, WA, USA; Janssen R&D, Titusville NJ, USA; Tufts Institute for Clinical Research and Health Policy Studies, US; Department of Biomedical Informatics, Columbia University Irving Medical Center, New York, NY 10032, USA; Nuffield Department of Clinical Neurosciences, University of Oxford, UK; Division of Respiratory and Critical Care Medicine, Department of Internal Medicine, Daegu Catholic University Medical Center, Daegu, Korea; University of Florida Health, Gainesville, FL, USA; Real World Solutions, IQVIA, Cambridge, MA, USA; Janssen R&D, Titusville NJ, USA, 2) Department of Medical Informatics, Erasmus University Medical Center, Rotterdam, The Netherlands; Division of Population Health and Genomics, University of Dundee, UK; Department of Medical Informatics & Clinical Epidemiology, Oregon Health & Science University, Portland, OR, USA; Fundació Institut Universitari per a la recerca a l’Atenció Primària de Salut Jordi Gol i Gurina (IDIAPJGol), Barcelona, Spain,; Centre for Statistics in Medicine, NDORMS, University of Oxford, UK; O’Brien Institute for Public Health, Faculty of Medicine, University of Calgary, Canada; Janssen R&D, Titusville NJ, USA; IOMED, Barcelona, Spain; University of Florida Health; Faculty of Medicine, Islamic University of Gaza, Palestine; Nanfang Hospital, Southern Medical University, Guangzhou, China; Department of Biomedical Informatics and Medical Education, University of Washington, Seattle, WA, USA; University of Florida; Department of Biomedical Sciences, Ajou University Graduate School of Medicine, Suwon, Korea; Director of Innovation and Digital Transformation, Hospital del Mar, Barcelona, Spain; Stanford University School of Medicine, Stanford, California, USA; Georgia State University, Department of Computer Science, Atlanta, GA, USA; Department of Infectious Diseases, Hospital del Mar, Institut Hospital del Mar d’Investigació Mèdica (IMIM), Universitat Autònoma de Barcelona. Universitat Pompeu Fabra, Barcelo; United States Agency for International Development, Washington, DC, USA; Nuffield Department of Orthopaedics, Rheumatology and Musculoskeletal Sciences, University of Oxford, Oxford, UK; Department of Biomedical Informatics, Columbia University Irving Medical Center, New York, NY 10032, USA, 2) New York-Presbyterian Hospital, 622 W 168 St, PH20 New York, NY 10032 USA; VINCI, VA Salt Lake City Health Care System, Salt Lake City, VA, & Division of Epidemiology, University of Utah, Salt Lake City, UT; Biomedical Big Data Center, Nanfang Hospital, Southern Medical University, Guangzhou, China; Data Science to Patient Value Program, University of Colorado Anschutz Medical Campus; Fundació Institut Universitari per a la recerca a l’Atenció Primària de Salut Jordi Gol i Gurina (IDIAPJGol), Barcelona, Spain; Department of Biomedical Informatics, Columbia University Irving Medical Center, New York, NY 10032, USA; DHC Technologies Co. Ltd, Beijing, China; VINCI, Tennessee Valley Healthcare System VA, Nashville, TN & Department of Biomedical Informatics, Vanderbilt University Medical Center, Nashville, TN; Real-World Evidence, TFS, Barcelona, Spain; Department of Medical Informatics & Clinical Epidemiology, Oregon Health & Science University, Portland, OR, USA; Stanford University; Massachusetts General Hospital, Harvard Medical School, Boston, MA, USA; Trial Form Support; Department of Biomedical Sciences, Ajou University Graduate School of Medicine, Suwon, Korea; Knight Cancer Institute, Oregon Health & Science University; Real World Solutions, IQVIA, Cambridge, MA, USA; VA Informatics and Computing Infrastructure, VA Salt Lake City Health Care System, Salt Lake City, UT, USA; Department of Biomedical Informatics, Ajou University School of Medicine, Suwon, South Korea; Department of Anesthesiology and Pain Medicine, Catholic University of Daegu, School of Medicine, Daegu, Korea; Fundació Institut Universitari per a la recerca a l’Atenció Primària de Salut Jordi Gol i Gurina (IDIAPJGol), Barcelona, Spain; Observational Health Data Analytics, Janssen Research and Development, Raritan, NJ, USA; University of Florida Health; Department of Biomedical Informatics, Columbia University Irving Medical Center, New York, NY 10032, USA; College of Engineering, The University of Arizona, Tucson, Arizona, USA; National Library of Medicine, National Institutes of Health, Bethesda, MD, USA; Nuffield Department of Orthopaedics, Rheumatology and Musculoskeletal Sciences, University of Oxford, Oxford, UK, 2) College of Medicine and Health, University of Exeter, St Luke’s Campus, E; Data Science to Patient Value Program, School of Medicine, University of Colorado Anschutz Medical Campus, Aurora, CO, USA; DHC Technologies Co. Ltd, Beijing, China; University of Florida Health; University of Florida Health; Department of Medical Informatics, Erasmus University Medical Center, Rotterdam, The Netherlands; Department of Biomedical Informatics, Columbia University Irving Medical Center, New York, NY 10032, USA, 2) New York-Presbyterian Hospital, 622 W 168 St, PH20 New York, NY 10032 USA; Janssen R&D; Department of Biostatistics, UCLA Fielding School of Public Health, University of California, Los Angeles

**Keywords:** COVID-19, OHDSI, OMOP CDM, hospital admission, descriptive epidemiology, real world data, real world evidence, open science

## Abstract

**Background:**

Routinely collected real world data (RWD) have great utility in aiding the novel coronavirus disease (COVID-19) pandemic response [1,2]. Here we present the international Observational Health Data Sciences and Informatics (OHDSI) [3] Characterizing Health Associated Risks, and Your Baseline Disease In SARS-COV-2 (CHARYBDIS) framework for standardisation and analysis of COVID-19 RWD.

**Methods:**

We conducted a descriptive cohort study using a federated network of data partners in the United States, Europe (the Netherlands, Spain, the UK, Germany, France and Italy) and Asia (South Korea and China). The study protocol and analytical package were released on 11^th^ June 2020 and are iteratively updated via GitHub [4].

**Findings:**

We identified three non-mutually exclusive cohorts of 4,537,153 individuals with a clinical *COVID-19 diagnosis or positive test, 886,193 hospitalized with COVID-19, and 113,627 hospitalized with COVID-19 requiring intensive services*. All comorbidities, symptoms, medications, and outcomes are described by cohort in aggregate counts, and are available in an interactive website: https:/data.ohdsi.org/Covid19CharacterizationCharybdis/.

**Interpretation:**

CHARYBDIS findings provide benchmarks that contribute to our understanding of COVID-19 progression, management and evolution over time. This can enable timely assessment of real-world outcomes of preventative and therapeutic options as they are introduced in clinical practice.

## Introduction

The World Health Organization (WHO) declared the coronavirus disease 2019 (COVID-19) pandemic on 11 March 2020 after 118,000 reported cases in over 110 countries [5]. By 2021, the number of COVID-19 cases has increased to over 90,000,000 globally, and as we write the death toll has reached 2 million [6]. Thousands of publications have attempted to aid our scientific understanding of this public health emergency [7,8].

Routinely collected real world data (RWD) are a powerful asset for an evolving pandemic response [1,2]. Each data source provides novel information, be it the geographic variability of COVID-19, the impact of varying government strategies to contain spread or the evolution of treatment protocols. With extensive heterogeneity in public health strategies and clinical care across the world [9], a large repeated multi-center study to describe disease across locations, practices, and populations, but that holds data analysis constant would go far in determining what factors impact observed differences.

RWD networks are vital in helping to understand the magnitude of the problem, and developing possibly mitigating strategies both globally and locally [10,11]. Here we present the global Observational Health Data Sciences and Informatics (OHDSI) community response to the COVID-19 pandemic [3]. Founded in 2015, the OHDSI data network enabled a rapid baseline understanding of COVID-19 in emerging hotspots (United States of America [USA], Spain and South Korea) [12]. Our work evolved into a systematic framework for analysing and reporting COVID-19 RWD that we call Characterizing Health Associated Risks, and Your Baseline Disease In SARS-COV-2 (CHARYBDIS).

CHARYBDIS offers multiple insights into COVID-19 clinical presentations, management and progression. We set out to continually describe baseline demographics, clinical characteristics, treatments received, and outcomes among individuals diagnosed and hospitalized with COVID-19 in actual practice settings in nine countries from three continents. Our body of research is a freely available, foundational result set that can provide benchmarks in how COVID-19 manifests over time including its inevitable evolution as we roll-out vaccines and treatments.

## Results

All comorbidities, presenting symptoms, medications and outcomes are reported by each cohort in aggregate counts, and are available in an interactive website: https:/data.ohdsi.org/Covid19CharacterizationCharybdis/.

### Patient characteristics

**Figure 1:**
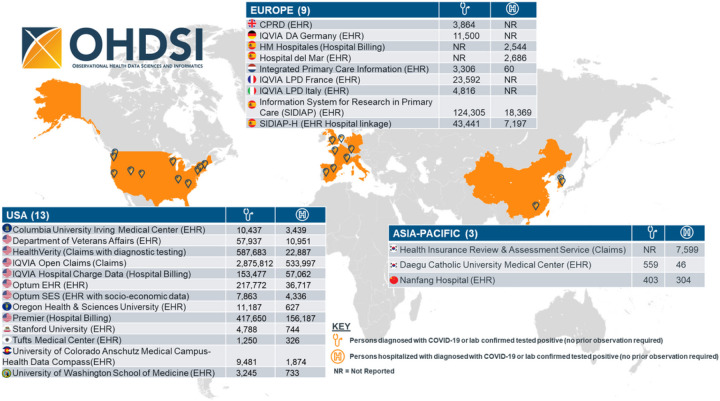
COVID-19 cases across the OHDSI COVID-19 network. Note: The designations employed and the presentation of the material on this map do not imply the expression of any opinion whatsoever on the part of Research Square concerning the legal status of any country, territory, city or area or of its authorities, or concerning the delimitation of its frontiers or boundaries. This map has been provided by the authors.

Overall, we identified three non-mutually exclusive cohorts of 4,537,153 individuals with a clinical *COVID-19 diagnosis or positive test, 886,193 hospitalized with COVID-19, and 113,627 hospitalized with COVID-19 requiring intensive services* (Figure 1). Of these, the cohorts including patients with the requirement of at least of 365 days before index: 3,279,518 with a clinical *COVID-19 diagnosis or laboratory positive test, 636,810 hospitalized with COVID-19, and 63,636 hospitalized with COVID-19 requiring intensive services* (Supplementary Tables 3 & 4).

### Geographic distribution

The USA data partners contributed 96% of the *diagnosed with COVID-19 cohorts*, including the single largest diagnosed cohort from IQVIA Open Claims (n=2,785,812). Europe contributed 4% of the *diagnosed with COVID-19* cohorts, owing the single largest regional diagnosed cohort to SIDIAP-Spain (n=124,305). Asia contributed less than 1% of *diagnosed with COVID-19 cohorts*, with the single largest regional diagnosed cohort contributed from Daegu Catholic University Medical Center (n=599).

### Demographic distribution

**Figure 2:**
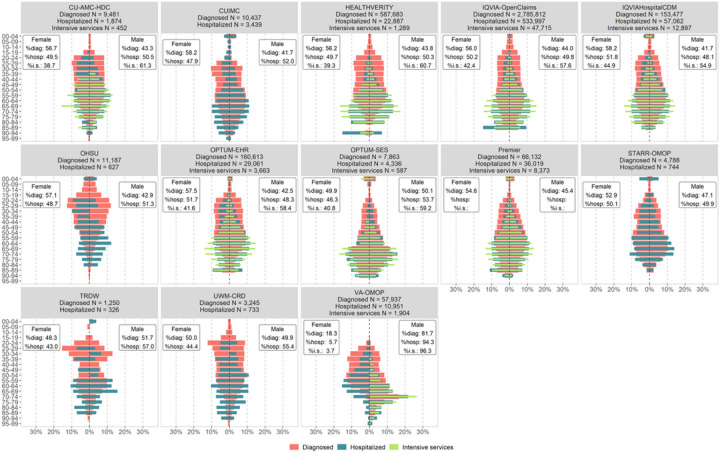
Distribution of diagnosed, hospitalized and requiring intensive services COVID-19 cases by age and sex across the OHDSI COVID-19 network in the United States NB: In each subplot, the x-axis represents what proportion of all women (left) and all men (right) fall in each age category. No prior observation period required in the cohorts shown in this figure. Cohorts must be >=140 people to be reported in this analysis. Abbreviations: diag: diagnosed; hosp: hospitalized; i.s.: hospitalized and requiring intensive services. Abbreviations: CU-AMC-HDC: U of Colorado Anschuz Medical Campus Health Data Compass; CUIMC: Columbia University Irving Medical Center; IQVIAHospitalCDM: IQVIA Hospital Charge Data Master; OHSU: Oregon Health and Science University; OPTUM-EHR: Optum© de-identified Electronic Health Record Dataset; OPTUM-SES: Optum® De-Identified Clinformatics® Data Mart Database – Socio-Economic Status (SES); STARR-OMOP: Stanford Medicine Research Data Repository; TRDW: Tufts MC Research Data Warehouse; UWM-CRD: UW Medicine COVID Research Dataset; VA-OMOP: Department of Veterans Affairs

In the USA, the proportion of diagnosed cases generally decreased with age, with most diagnosed cases being within the 25 to 60 age group. The proportion of cases hospitalized and intensive services increased with age, with the highest proportions of cases of hospitalized, or intensive cases in the 60 to 80 year age group (Figure 2). A slightly higher proportion of women were diagnosed than men but a greater proportion of men were hospitalized (and where available, required intensive services) than women in the USA databases. In Europe, databases captured diagnosed or hospitalised cohorts but had limited information on intensive services. In Europe, databases capturing hospitalized cases (HMAR, HM-Hospitales, SIDIAP, and SIDIAP-H) showed a similar trend to the USA databases in that there was a higher proportion of men were hospitalized than women (Supplementary Figure 1). Unlike the USA and European databases, there was also a higher proportion of women in hospitalized cases in the South Korean database (HIRA). Age-wise trends in the European and Asian databases were similar to those in the USA databases, in that the bulk of the diagnosed cases were in the 25 to 60 year age group, whilst the majority of the hospitalized cases were in the 60 to 80 year age group (Supplementary Figure 1).

### Comorbidities

Overall, the proportion of patients with type 2 diabetes mellitus, hypertension, chronic kidney disease, end stage renal disease, heart disease, malignant neoplasm, obesity, dementia, auto-immune condition, chronic obstructive pulmonary disease (COPD), and asthma was higher in the hospitalised cohort as compared to the diagnosed (Tables 1 and 2). Data on tuberculosis, human immunodeficiency viruses (HIV), and hepatitis C infections were sparse, and where available the proportions were generally low (<=1%). In the US databases, the proportion of pregnant women was generally higher in the hospitalised cohort than in the diagnosed, but not so in two European databases (HM and SIDIAP). The remaining five European and one of the Asian databases had data on pregnant women only in the hospitalised cohort, the proportion of which was < 2%.

### Other analyses

Dyspnea, cough, and fever were the most common symptoms in diagnosed and hospitalized cohorts (Supplementary Table 5). Where recorded, the proportion of dyspnea and malaise/fatigue was consistently higher in the hospitalised cohort as compared to the diagnosed.

Anosmia/hyposmia/dysgeusia was present in less than 1% individuals in all but one database and more common in the diagnosed than the hospitalised cohorts.

We further described a total of 19,222 conditions and 2,973 medications registered during the year prior to the index date (Supplementary Figure 2). The same information is also described for 30 days prior to the index date, at index date, or during the first 30 days after index date (this can be explored in detail at https:/data.ohdsi.org/Covid19CharacterizationCharybdis/).

## Discussion

### Summary of key findings

We described characteristics of 4,537,153 individuals with a clinical *COVID-19 diagnosis or positive test, 886,193 hospitalized with COVID-19, and 113,627 hospitalized with COVID-19 requiring intensive services* from 9 countries. Up to 22,200 unique aggregate characteristics have been produced across databases, with all made publicly available in an accompanying website. The cumulative evidence obtained from different regions and at different points in the pandemic can guide in 1) better patient characterization and stratification, 2) identifying areas of gap in knowledge/evidence, and 3) generating hypotheses for future research.

### Findings in context

In April 2020, the National COVID Cohort Collaborative (N3C) chose the OMOP CDM as the data model for centralizing patient-level data to study patterns in COVID-19 patients [20]. This network has over 80 participating institutions and is enabling many US institutions in adoption of common data models in COVID-19 research. This program has two major differences: 1) data are limited to US only sites and 2) the centralized data approach requires significant programmatic oversight. In contrast to this and other notable RWD initiatives, CHARYBDIS uses an existing decentralized network, open to all, with no requirement to move patient-level data [21]. This enables the opportunity to integrate results from regions within more restrictive data sharing policies, such as Europe and Asia.

The Consortium for Clinical Characterization of COVID-19 by EHR (4CE), is another multi-site data-sharing collaborative of 342 hospitals in the US and in Europe, utilizing i2b2 or OMOP data models [22]. Despite its extensive footprint, 4CE cohorts remain smaller than the scope of CHARYBDIS with only 36,447 patients with COVID-19 as of August 2020 [22]. Even with cohort overlap, our work to date with CHARYBDIS is substantial spanning 4.5 million COVID-19 patients across three continents.

The “tragic data gap” undermining response to the pandemic [23] is effected by inadequate utilization of and access to high-quality RWD. Large scale initiatives like CHARYBDIS can offer critical infrastructure for mobilizing simple descriptive epidemiological studies that are fundamentally important in tracking the evolution and ultimate management of this pandemic. Our findings can help proivde context on where to direct future funding and carry out additional research. The information generated from CHARYBDIS can inform the response to the pandemic, including both public health restrictions (non-pharmacological interventions) and vaccination strategies worldwide. As we continue our research, we are also actively curating relationships with data partners to drive inpatient-outpatient linkages and understand COVID-19 patient trajectories across care settings.

### Study strengths

Our study has several strengths. This study is unique in its approach to characterizing COVID-19 cases across an international network of healthcare systems with varied policies enacted to combat this pandemic. This allows better understanding of the implications of the pandemic for different countries and regions, in the context of an international comparison. Particularly, it provides visibility into the inherent variability of patient characteristics across healthcare settings. This study is the most comprehensive federated network of healthcare sites in the world, creating the single largest cohort study on diagnosed and hospitalized COVID-19 cases to date. The large, diverse sample size allows also for the identification of populations of great interest, including children and adolescents, pregnant women, patients with a history of cancer, or patients with HIV, who were also infected with COVID-19, and who will be the focus of in-depth future investigations.

### Study limitations

We recognize there are limitations in our approach. First, this study is descriptive in nature and was not designed for causal inference. The observed differences between groups (e.g. diagnosed versus hospitalized) should therefore not be interpreted as causal effects. Answering causal questions is especially difficult in COVID-19 because of the varying processes by which patients were screened, tested, admitted, and treated; the critical importance of knowing the exact timing of treatments and outcomes in severe cases; and the lack of appropriate comparison groups. Simple multivariable models by themselves will not sufficiently address bias for multiple questions and were purposely not applied here. This study was carried out using data recorded in routine clinical practice and based on electronic health records (EHRs) and/or claims data. The analysed data are therefore expected to be incomplete in some respects and may have erroneous entries, leading to potential misclassification. We have selectively reported database-specific outcomes to minimise the impact of incompleteness. Additionally, the under-reporting of symptoms observed in these data is a key finding of this study, and should be taken into consideration in previous and future similar reports from ‘real world’ cohorts. Differential reporting in different databases is likely a function of differential coding practice as well as of variability in disease severity, with milder/less symptomatic cases more likely presenting in outpatient and primary care EHR, and more severe ones in hospital databases. Finally, the current result submissions are prejudiced to data in the initial wave of COVID-19 cases and may not be representative of the data during subsequent waves. We currently lack data partners in low to middle income countries and are actively building collaborations in these areas. As data are accumulated over time, future updates of the results will provide the opportunity to study more recent cohorts of COVID-19 patients, who seem to have a better prognosis overall compared to those diagnosed in the first half of the year.

## Conclusions

We present the foundation for an epidemiological framework to perform large scale characterization of the presentation, management, and outcomes of COVID-19 as observed in actual practice settings worldwide. We have characterized the natural history of over 4.5 million COVID-19 patients from the USA, 6 European countries and 2 Asian countries. This work allows deep phenotyping of COVID-19, serving as a repeatable, reproducible method to capture the evolving natural history of this novel coronavirus and can be extended to future pandemics. Leveraging our global federated network to corroborate single center findings can provide context to national database findings in the presence of regional variability in COVID-19 policies. This effort provides critical infrastructure for mobilizing descriptive studies that are fundamentally important in tracking the evolution and ultimate management of this pandemic.

## Methods

### Study design, setting and data sources

We conducted a descriptive cohort study using a federated network of data partners in the USA, Europe (the Netherlands, Spain, the UK, Germany, France and Italy) and Asia (South Korea and China). We required each data partner to map their source system to the Observational Medical Outcomes Partnership (OMOP) common data model (CDM) [13–15]. The use of a CDM ensured shared conventions, including consistent representation of clinical terms across coding systems. We deployed a common data quality tool for repeated assessment and monitoring the adherence to conventions across the network [16,17]. We ensured reproducibility by using the same package of analytical code for all contributing data partners [18].

The study protocol and analytical package were released on 11 June 2020 and iterative updates have continued to be released via GitHub: https:/github.com/ohdsi-studies/Covid19CharacterizationCharybdis [4]. As of February 2021, 26 databases have contributed to the CHARYBDIS study (Supplementary Table 1). Contributing institutes ranged from major academic medical centers to small community hospitals from across three continents. While most data were captured from March to June 2020, a subset of data partners submitted updates through October 2020. Two sites report data through December 2020. Additional updates are expected as data partners refresh their OMOP CDM data. Prior to performing these analyses, all the data partners obtained Institutional Review Board (IRB) or equivalent governance approval. Each data partner executed the study package locally on their OMOP CDM. Only aggregate results from each database were publicly shared. Minimum cell sizes were determined by institutional protocols. All data partners consented to the external sharing of the result set on data.ohdsi.org.

### Study population and follow-up

We focused on three non-mutually exclusive COVID-19 cohorts: i) *diagnosed with COVID-19* (a positive SARS-CoV-2 laboratory test or clinical diagnosis of COVID-19 - earliest event served as the index date); ii) *hospitalized with COVID-19* and; iii) *hospitalized with COVID-19 and requiring intensive services*. The codes used to identify cohorts and more detail on the definitions of the above cohorts can be found in Supplementary Table 2. These cohorts were generated both with a requirement of at least 365 days of data availability prior to the index date, and without any requirement for prior observation time. Datamarts created specifically for COVID-19 tracking may be unable to support extensive lookback periods and thus, we used multiple definitions to ensure inclusiveness in our approach. Cohorts were followed from their cohort-specific index date to the earliest of death, end of the observation period, and up to 30 days post-index.

### Stratifications

Each cohort was analyzed by the overall study population and stratified by additional available characteristics including: follow-up time; socio-demographics, baseline comorbidities, pregnancy status (yes/no), and flu-like symptom episodes (yes/no). Detailed definitions of each stratification are available in Supplementary Table 2.

### Baseline characteristics, symptoms, medication use and outcomes of interest

Information on socio-demographics was identified at or before baseline (index date). All conditions, symptoms and medications were identified and described at four different time intervals (1 year prior, 30 days prior, at index and up to 30 days after index). The definition of each symptom and outcome is provided in Supplementary Table 2.

### Statistical analysis

We built this analysis using Health Analytics Data-to-Evidence Suite (HADES), a set of open source R packages for large scale analytics [19]. Proportions, standard deviations (SD), and standardized mean differences (SMD) within each subgroup were tabulated as pre-specified in our study protocol. This analysis was descriptive in nature with no causal inference intended. Only cohorts or stratified sub-cohorts with a minimum sample size of 140 subjects were characterized. This cut-off was deemed necessary to estimate with sufficient precision the prevalence of a previous condition or 30-day risk of an outcome affecting >=10% of the study population. SMDs were plotted in Manhattan-style plots, a type of scatter plot designed to visualize large data with a distribution of higher-magnitude values. Scatter plots were also created to compare the described conditions, symptoms and demographics of patients diagnosed (Y axis) to those hospitalized (X axis) with COVID-19.

## Supplementary Material

Supplement

Supplement
