# Supplementary material for: Unraveling COVID-19: a large-scale characterization of 4.5 million COVID-19 cases using CHARYBDIS
Source: Res Sq. 2021 Mar 1:rs.3.rs-279400. Preprint. [Version 1] doi: 10.21203/rs.3.rs-279400/v1 (PMC7941629; doi:10.21203/rs.3.rs-279400/v1)
Supplement: Supplement [file 9a3d13d617f019ad05c41fb1.pdf]

Unraveling COVID-19: a large-scale characterization of 4.5 million COVID-19 cases using CHARYBDIS

SUPPLEMENTAL MATERIAL

Supplementary Table 1. Overview of participating databases mapped to the OMOP-CDM.

Database name	Setting	Patient Count (in Millions)	Data History	Data capture process and short database description
HealthVerity	Commercially insured – USA	Not Reported	Not Reported	HealthVerity derived data set contains de-identified patient information with an antibody and/or diagnostic test for COVID-19 linked to all available Medical Claims and Pharmacy Data from select private data providers participating in the HealthVerity marketplace.
Premier	Hospital billing – USA	Not Reported	Not Reported	The Premier Healthcare Database contains complete clinical coding, hospital cost, and patient billing data from approximately 700 hospitals throughout the United States representing 20% of inpatient hospital stays. Premier collects data from participating hospitals in its health care alliance. The Premier health care alliance was formed for hospitals to share knowledge, improve patient safety, and reduce risks. Participation in the Premier health care alliance is voluntary. Although the database excludes federally funded hospitals (e.g., Veterans Affairs), the hospitals included are nationally representative based on bed size, geographic region, location (urban/rural) and teaching hospital status. The database contains a date-stamped log of all billed items by cost-accounting department including medications; laboratory, diagnostic, and therapeutic services; and primary and secondary diagnoses for each patient's hospitalization.
Clinical Practice Research Datalink (CPRD)	Primary care – UK	Not Reported	Not Reported	The Clinical Practice Research Datalink (CPRD) is a governmental, not-for-profit research service, jointly funded by the NHS National Institute for Health Research (NIHR) and the Medicines and Healthcare products Regulatory Agency (MHRA), a part of the Department of Health, United Kingdom (UK). CPRD consists of data collected from UK primary care for all ages. This includes conditions, observations, measurements, and procedures that the general practitioner is made aware of in additional to any prescriptions as prescribed by the general practitioner. In addition to primary care, there are also linked secondary care records for a small number of people. The major data elements contained within this database are outpatient prescriptions given by the general practitioner (coded with Multilex codes) and outpatient clinical, referral, immunization or test events that the general practitioner knows about (coded in Read or ICD10 or LOINC codes). The database also contains the patients' year of births and any date of deaths.

Daegu Catholic University Medical Center	Hospital EHR – South Korea	8,841	Jan, 2005-Jul, 2020	A teaching hospital in Daegu, South Korea, covered by Federated E-health Big Data for Evidence Renovation Network (FEEDER-NET). It contains the data from patients in the early days of COVID-19 endemic in South Korea.
Hospital del Mar (HMAR)	Hospital EHR – Barcelona, Spain	0.6M	2000-Late 2020	Anonymized data from the Electronic Medical Records from Hospital del Mar (Barcelona, Spain). Hospital belonging to the Spanish National Health System (public), attending the Eastern area of Barcelona City. Includes hospital data collected routinely in the clinical practice, both structured and unstructured information, extracted using a free text analysis tool (with natural language processing): Inpatient (hospital) care, Outpatient specialist care, Emergency Room Visits and partial information from other settings like primary care and pharmacy care present in free text notes from EMRs. All subjects with at least one healthcare encounter with the Hospital within approximately the last 20 years are included (approximately 0.6 M subjects, with more than 5 M hospitalizations/visits). Hospital del Mar data are made available through collaboration with TFS / IOMED.
Health Insurance Review & Assessment Service	Claims – South Korea	Not Reported	Not Reported	National claim data from a single insurance service from South Korea. It contains the observational medical records (including both inpatient and outpatient) of a patient while they are qualified to get the national medical insurance.
HM Hospitals	Hospital EHR – Spain	Not Reported	Not Reported	Hospital de Madrid (HM) Hospitals data are made available through partnership with IDIAPJGol. The HM Hospitals database covers in-patient care delivered across a network of 17 private hospitals in Spain between 1st of March and 24th of April 2020. HM Hospitals database covers more than 2300 confirmed COVID-19 cases, and all in-patient hospital care, including the data of admission, conditions, procedures and medicines dispensed in hospital, date of discharge, and date of known death or date of end of follow-up in the database.
Nanfang Hospital COVID-19 Research Database (NFHCRD)	Hospital EHR – Hubei, China	Not Reported	Not Reported	The clinical data warehouse of The People's Hospital of HongHu，Hubei，China，based on its current and previous electronic health record systems, with data spanning over 4 months and including over 400 patients.
Optum EHR	National EHR – USA	Not Reported	Not Reported	Optum© de-identified Electronic Health Record Dataset represents Humedica's Electronic Health Record data a medical records database for patients receiving a COVID-19 diagnosis record or lab test for SARS-CoV-2. The medical record data includes clinical information, inclusive of prescriptions as prescribed and administered, lab

				results, vital signs, body measurements, diagnoses, procedures, and information derived from clinical Notes using Natural Language Processing (NLP).
Information System for Research in Primary Care – Hospitalization Linked Data (SIDIAP-H)	Linked Primary Care EHR to Hospital EHR – Catalonia, Spain	2M	Jan 1st 2006-June 6th 2020	The Information System for Research in Primary Care (SIDIAP; www.sidiap.org) is a primary care records database from Catalonia, North-East Spain. The SIDIAP-H subset of the database includes around 2 million people out of the total 7 million in SIDIAP that are registered in primary care practices with linked hospital inpatient data available as obtained from the Catalan Institute of Health hospitals. Healthcare is universal and tax-payer funded in the region, and primary care physicians are gatekeepers for all care and responsible for repeat prescriptions.
Information System for Research in Primary Care (SIDIAP)	Primary Care EHR - Catalonia, Spain	7.8M	Jan 1st 2006-June 6th 2020	The Information System for Research in Primary Care (SIDIAP; www.sidiap.org) is a primary care records database that covers approximately 7 million people, equivalent to an 80% of the population of Catalonia, North-East Spain. Healthcare is universal and tax-payer funded in the region, and primary care physicians are gatekeepers for all care and responsible for repeat prescriptions.
STARR-OMOP	Hospital EHR – California, USA	Not Reported	Not Reported	STAnford medicine Research data Repository, a clinical data warehouse containing live Epic data from Stanford Health Care, the Stanford Children's Hospital, the University Healthcare Alliance and Packard Children's Health Alliance clinics and other auxiliary data from Hospital applications such as radiology PACS. STARR platform is developed and operated by Stanford Medicine Research IT team and is made possible by Stanford School of Medicine Research Office. (Datta et al. 2020)
Tufts MC Research Data Warehouse (TRDW)	Hospital EHR - Massachusetts, USA	Not Reported	Not Reported	Electronic medical record data on approximately 1 million patients who recived care beginning in 2006 at Tufts Medical Center (TMC). TMC is an academic medical center that includes Tuft Medical Center's main downtown Boston hospital for adult patients, the Floating Hospital for Children, and associated primary and specialty care clinics. TRDW contains TMC's EHR data fused with data on the same patients from TMC's CoC accredited tumor registry, its oncology EHR, and death data from the Massachusetts State Registry of Vital Statistics. EHR data streams ingested into TRDW include controlled vocabulary data on all domains except cost, and select free text sources and devices.
Department of Veterans Affairs (VA OMOP)	Hospital EHR - USA	Not Reported	Not Reported	VA OMOP data reflects the national Department of Veterans Affairs health care system, which is the largest integrated provider of medical and mental health services in the United States. Care is provided at 170 VA Medical Centers and 1,063 outpatient sites serving more than 9 million enrolled Veterans each year.

Integrated Primary Care Information (IPCI)	Primary Care - Netherlands	Not Reported	Not Reported	The Integrated Primary Care Information (IPCI) database is a Dutch database containing the medical records of more than 2.5 million patients provided by more than 600 GPs geographically spread over the Netherlands. In the Netherlands, all citizens are registered with a GP practice which acts as a gatekeeper in a two-way exchange of information with secondary care.
IQVIA Open Claims	Commercial Claims – USA	>200M	2013-October 2020	A United States database of open, pre-adjudicated claims from January 2013 to May 2020. Data are reported at anonymized patient level collected from office-based physicians and specialists via office management software and clearinghouse switch sources for the purpose of reimbursement. A subset of medical claims data have adjudicated claims.
Columbia University Irving Medical Center	Hospital EHR – New York, USA			The clinical data warehouse of NewYork-Presbyterian Hospital/Columbia University Irving Medical Center, New York, NY, based on its current and previous electronic health record systems, with data spanning over 30 years and including over 6 million patients
IQVIA Longitudinal Patient Data France	Primary Care – France	7.8M	1994-June 2020	IQVIA LPD France is a computerised network of physicians including GPs who contribute to a centralised database of anonymised patient EMR. Currently, >1200 GPs from 400 practices are contributing to the database covering 7.8M patients in France. The database covers a time period from 1994 through the present. Observation time is defined by the first and last consultation dates. Drug information is derived from GP prescriptions. Drugs obtained over the counter by the patient outside the prescription system are not reported.
IQVIA Longitudinal Patient Data Italy	Primary Care – Italy	2M	2004-July 2020	LPD Italy is comprised of anonymised patient records collected from software used by GPs during an office visit to document patients' clinical records. Data coverage includes over 2M patient records with at least one visit and 119.5M prescription orders across 900 GP practices. Dates of service include from 2004 through present. Observation time is defined by the first and last consultation dates. Drugs are captured as prescription records with product
IQVIA Disease Analyser Germany	Primary Care – Germany	34M	1992-September 2020	IQVIA LPD DA Germany is collected from extracts of patient management software used by GPs and specialists practicing in ambulatory care settings. Data coverage includes more than 34M distinct person records out of at total population of 80M (42.5%) in the country and collected from 2,734 providers. Dates of service include from 1992 through September 2020.
Oregon Health & Science University	Hospital EHR – Oregon, USA	3M	2010 – January 2021	Oregon Health & Science University's Research Data Warehouse that contains over 3 million patients over more than 10 years; the Oregon Clinical & Translational Research Institute.

UW Medicine COVID Research Dataset (UWM-CRD)	Hospital EHR – Washington, USA	5M	2010-Late 2020	The dataset includes UW Medicine patients who have been tested for COVID-19 that have information within the UW Medicine Electronic Health Record. The dataset is a subgroup of the non-OMOP clinical data warehouse of University of Washington Medical Center, comprised of Harborview Medical Center, UW Medical Center - Montlake, and UW Medical Center - Northwest in Seattle WA, and is based on its current electronic health record systems, with data spanning over 10 years and including roughly 5 million patients.

Unraveling COVID-19: a large-scale characterization of 4.5 million COVID-19 cases using CHARYBDIS

Supplementary Figure 1. Distribution of diagnosed, hospitalized and requiring intensive services COVID-19 cases by age and sex across the OHDSI COVID-19 network.

NB: In each subplot, the x-axis represents what proportion of all women (left) and all men (right) fall in each age category. No prior observation period required in the cohorts shown in this figure. Cohorts must be >=140 people to be reported in this analysis. Abbreviations: diag: diagnosed; hosp: hospitalized; i.s.: hospitalized and requiring intensive services.

A. **Europe**

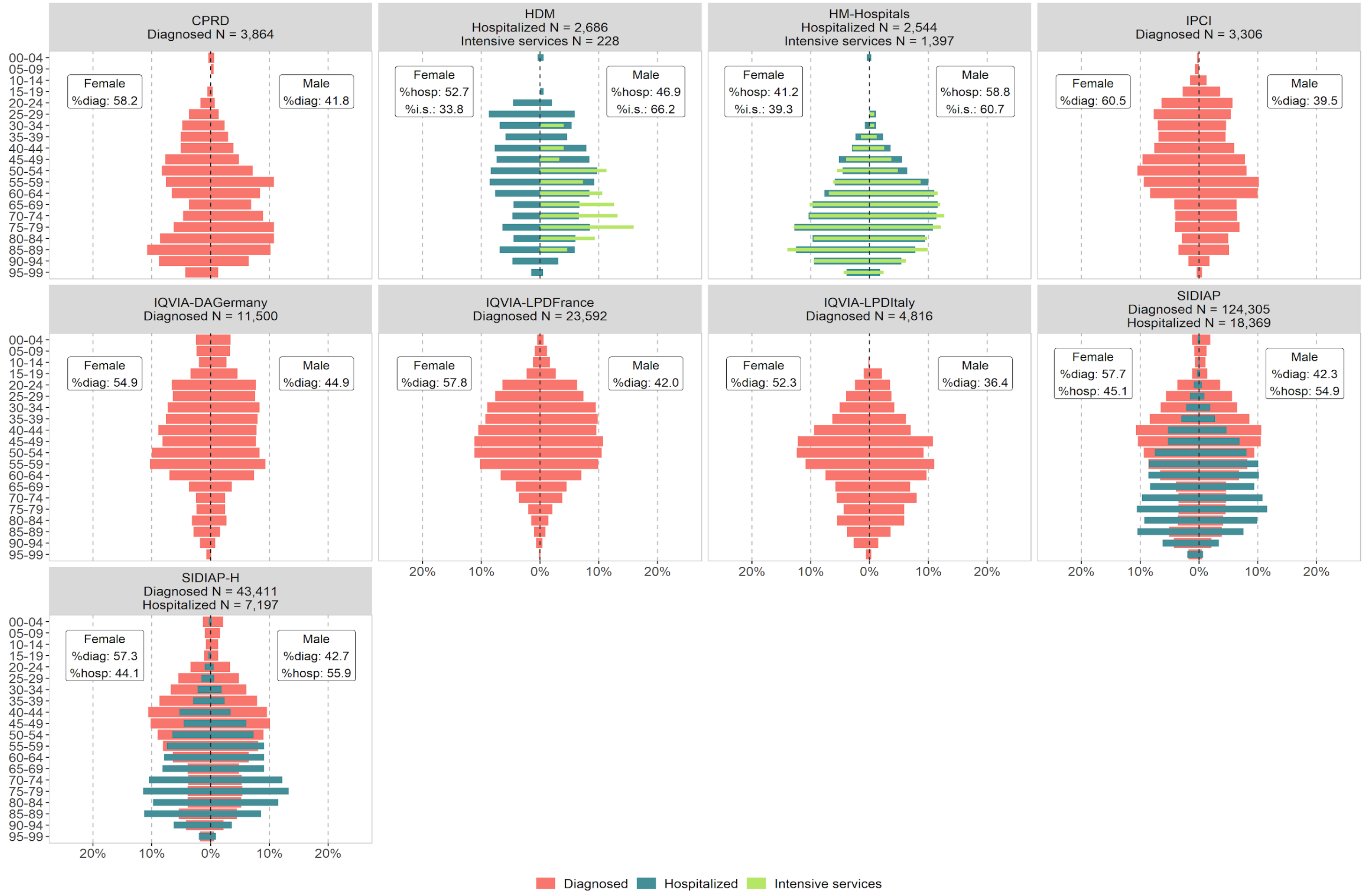

Abbreviations: CPRD: Clinical Practice Research Datalink – Outpatient Setting; HDM/HMAR: Hospital del Mar – Inpatient Setting; HM Hospitals – Inpatient Setting; IPCI: Integrated Primary Care Information – Outpatient Setting; IQVIA

Unraveling COVID-19: a large-scale characterization of 4.5 million COVID-19 cases using CHARYBDIS

Disease Analyser Germany -Outpatient Setting; IQVA Longitudinal Patient Data France – Outpatient Setting; IQVIA Longitudinal Patient Data Italy – Outpatient Setting; SIDIAP: Information System for Research in Primary Care – Outpatient Setting; SIDIAP-H: Information System for Research in Primary Care with Hospital Linkage

B. Asia

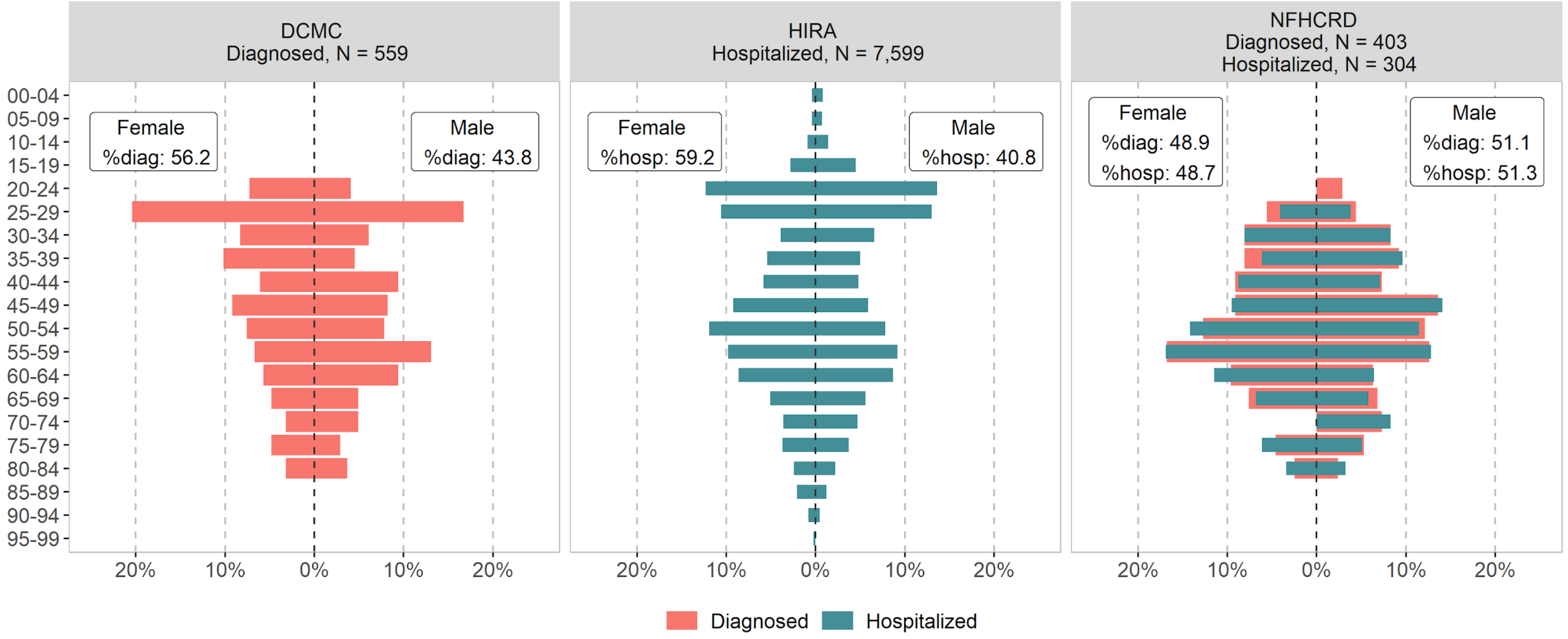

Abbreviations: DCMC: Daegu Catholic University Medical Center (South Korea) – Outpatient Setting; HIRA: Health Insurance Review & Assessment Service (South Korea) – Inpatient Setting; NFHCRD: Nanfang Hospital COVID-19 Research Database (NFHCRD) – Inpatient/Outpatient Setting

Unraveling COVID-19: a large-scale characterization of 4.5 million COVID-19 cases using CHARYBDIS

Supplementary Table 2. Cohort definitions

How to access: atlas.ohdsi.org requires you to log into a G-Suite affiliated account to see the library of Cohort Definitions. To access the Definition URL, click on the link and proceed to Log-In (upper right corner). The Log-In can be any G-Suite affiliated account. When you are finished, it will bring you back to the ATLAS home page. You can then click on the Definition URL again and it should allow you to see the contents of that definition.

Cohort name	Definition URL
Abortion	https://atlas.ohdsi.org/#/cohortdefinition/263
Acute kidney injury (AKI) diagnosis during hospitalization	https://atlas.ohdsi.org/#/cohortdefinition/276
Acute kidney injury (AKI) using diagnosis codes and change in measurements during hospitalization	https://github.com/ohdsi-studies/Covid19CharacterizationCharybdis/tree/master/inst/sql/sql_server/201.sql
Acute myocardial infarction events	https://atlas.ohdsi.org/#/cohortdefinition/239
Acute pancreatitis events	https://atlas.ohdsi.org/#/cohortdefinition/247
Acute Respiratory Distress syndrome (ARDS) during hospitalization	https://atlas.ohdsi.org/#/cohortdefinition/278
Angina during hospitalization	https://atlas.ohdsi.org/#/cohortdefinition/250
Anosmia OR Hyposmia OR Dysgeusia	https://atlas.ohdsi.org/#/cohortdefinition/283
Asthma/COPD Step 1	https://atlas.ohdsi.org/#/cohortdefinition/216
Asthma/COPD Step 2	https://atlas.ohdsi.org/#/cohortdefinition/215
Asthma/COPD Step 3	https://atlas.ohdsi.org/#/cohortdefinition/214
Bleeding during hospitalization	https://atlas.ohdsi.org/#/cohortdefinition/238
Bradycardia or heart block during hospitalization	https://atlas.ohdsi.org/#/cohortdefinition/271
Cardiac arrhythmia during hospitalization	https://atlas.ohdsi.org/#/cohortdefinition/248
Cardiovascular-related mortality	https://atlas.ohdsi.org/#/cohortdefinition/244
Cesarean section	https://atlas.ohdsi.org/#/cohortdefinition/260
Cough	https://atlas.ohdsi.org/#/cohortdefinition/287
Death	https://atlas.ohdsi.org/#/cohortdefinition/96
Deep vein thrombosis events	https://atlas.ohdsi.org/#/cohortdefinition/273
dialysis during hospitalization	https://atlas.ohdsi.org/#/cohortdefinition/252
Discharge from hospitalization	https://github.com/ohdsi-studies/Covid19CharacterizationCharybdis/tree/master/inst/sql/sql_server/202.sql
Dyspnea	https://atlas.ohdsi.org/#/cohortdefinition/284
Eclampsia and pre-eclampsia	https://atlas.ohdsi.org/#/cohortdefinition/289

ECMO during hospitalization	https://atlas.ohdsi.org/#/cohortdefinition/253
Fetal growth restriction	https://atlas.ohdsi.org/#/cohortdefinition/261
Fever	https://atlas.ohdsi.org/#/cohortdefinition/288
Flu-like symptom episodes	https://atlas.ohdsi.org/#/cohortdefinition/290
Gastrointestinal bleeding events	https://atlas.ohdsi.org/#/cohortdefinition/245
Gestational diabetes	https://atlas.ohdsi.org/#/cohortdefinition/225
Heart failure during hospitalization	https://atlas.ohdsi.org/#/cohortdefinition/272
Hemorrhagic stroke (intracerebral bleeding) events	https://atlas.ohdsi.org/#/cohortdefinition/240
Hospitalization episodes	https://atlas.ohdsi.org/#/cohortdefinition/280
Hospitalization for Asthma	https://atlas.ohdsi.org/#/cohortdefinition/259
Hospitalization for COPD	https://atlas.ohdsi.org/#/cohortdefinition/258
Hospitalization for psychosis	https://atlas.ohdsi.org/#/cohortdefinition/236
Incident depression with no prior treatment and no mania/psychoses	https://atlas.ohdsi.org/#/cohortdefinition/237
intensive services during hospitalization	https://atlas.ohdsi.org/#/cohortdefinition/316
Ischemic stroke events	https://atlas.ohdsi.org/#/cohortdefinition/241
Livebirth Delivery	https://atlas.ohdsi.org/#/cohortdefinition/267
Livebirth excluding preterm and post term delivery	https://atlas.ohdsi.org/#/cohortdefinition/266
Livebirth Post term Delivery	https://atlas.ohdsi.org/#/cohortdefinition/265
Livebirth Preterm Delivery	https://atlas.ohdsi.org/#/cohortdefinition/264
Malaise or fatigue	https://atlas.ohdsi.org/#/cohortdefinition/285
mechanical ventilation during hospitalization	https://atlas.ohdsi.org/#/cohortdefinition/318
Multi-system inflammatory syndrome (Kawasaki disease or toxic shock syndrome)	https://atlas.ohdsi.org/#/cohortdefinition/234
Myalgia	https://atlas.ohdsi.org/#/cohortdefinition/286
Persons hospitalized and requiring intensive services with a COVID-19 diagnosis record or a SARS-CoV-2 positive test with at least 365d prior observation	https://atlas.ohdsi.org/#/cohortdefinition/305
Persons hospitalized and requiring intensive services with a COVID-19 diagnosis record or a SARS-CoV-2 positive test with no required prior observation	https://atlas.ohdsi.org/#/cohortdefinition/308
Persons hospitalized and requiring intensive services with a SARS-CoV-2 positive test with at least 365d prior observation	https://atlas.ohdsi.org/#/cohortdefinition/306

Persons hospitalized and requiring intensive services with a SARS-CoV-2 positive test with no required prior observation	https://atlas.ohdsi.org/#/cohortdefinition/309
Persons hospitalized with a COVID-19 diagnosis record or a SARS-CoV-2 positive test with at least 365d prior observation	https://atlas.ohdsi.org/#/cohortdefinition/197
Persons hospitalized with a COVID-19 diagnosis record or a SARS-CoV-2 positive test with no required prior observation	https://atlas.ohdsi.org/#/cohortdefinition/198
Persons hospitalized with a SARS-CoV-2 positive test with at least 365d prior observation	https://atlas.ohdsi.org/#/cohortdefinition/191
Persons hospitalized with a SARS-CoV-2 positive test with no required prior observation	https://atlas.ohdsi.org/#/cohortdefinition/194
Persons tested for SARS-CoV-2 with at least 365d prior observation	https://atlas.ohdsi.org/#/cohortdefinition/206
Persons tested for SARS-CoV-2 with no required prior observation	https://atlas.ohdsi.org/#/cohortdefinition/205
Persons tested positive for SARS-CoV-2 with at least 365d prior observation	https://atlas.ohdsi.org/#/cohortdefinition/204
Persons tested positive for SARS-CoV-2 with no required prior observation	https://atlas.ohdsi.org/#/cohortdefinition/203
Persons tested with a COVID-19 diagnosis record or a SARS-CoV-2 positive test with at least 365d prior observation	https://atlas.ohdsi.org/#/cohortdefinition/202
Persons tested with a COVID-19 diagnosis record or a SARS-CoV-2 positive test with no required prior observation	https://atlas.ohdsi.org/#/cohortdefinition/201
Persons with a COVID-19 diagnosis or a SARS-CoV-2 positive test with at least 365d prior observation	https://atlas.ohdsi.org/#/cohortdefinition/200
Persons with a COVID-19 diagnosis or a SARS-CoV-2 positive test with no required prior observation	https://atlas.ohdsi.org/#/cohortdefinition/199
Persons with additional testing for SARS-Cov-2 (prior test >=1d before test)	https://atlas.ohdsi.org/#/cohortdefinition/282
Persons with additional testing for SARS-Cov-2 (prior test >=5d before test)	https://atlas.ohdsi.org/#/cohortdefinition/281
Persons with chest pain or angina	https://atlas.ohdsi.org/#/cohortdefinition/251
Persons with hepatic failure	https://atlas.ohdsi.org/#/cohortdefinition/249
Pneumonia during hospitalization	https://atlas.ohdsi.org/#/cohortdefinition/279
Pneumonia episodes	https://atlas.ohdsi.org/#/cohortdefinition/257
Pregnant women	https://atlas.ohdsi.org/#/cohortdefinition/233
Premature Rupture of Membranes	https://atlas.ohdsi.org/#/cohortdefinition/262
Prevalent Asthma or Chronic obstructive pulmonary disease (COPD)	https://atlas.ohdsi.org/#/cohortdefinition/217
Prevalent Asthma without COPD	https://atlas.ohdsi.org/#/cohortdefinition/218
Prevalent Autoimmune condition	https://atlas.ohdsi.org/#/cohortdefinition/220

Prevalent chronic kidney disease	https://atlas.ohdsi.org/#/cohortdefinition/228
Prevalent chronic kidney disease broad	https://atlas.ohdsi.org/#/cohortdefinition/312
Prevalent chronic obstructive pulmonary disease (COPD) without asthma	https://atlas.ohdsi.org/#/cohortdefinition/219
Prevalent Dementia	https://atlas.ohdsi.org/#/cohortdefinition/226
Prevalent end stage renal disease	https://atlas.ohdsi.org/#/cohortdefinition/232
Prevalent end stage renal disease broad	https://atlas.ohdsi.org/#/cohortdefinition/313
Prevalent heart disease	https://atlas.ohdsi.org/#/cohortdefinition/231
Prevalent Hepatitis C	https://atlas.ohdsi.org/#/cohortdefinition/230
Prevalent Human immunodeficiency virus infection	https://atlas.ohdsi.org/#/cohortdefinition/229
Prevalent Human immunodeficiency virus infection broad	https://atlas.ohdsi.org/#/cohortdefinition/314
Prevalent hypertension	https://atlas.ohdsi.org/#/cohortdefinition/227
Prevalent malignant neoplasm excluding non-melanoma skin cancer (note: referred to as “Prior cancer” in tables for space purposes)	https://atlas.ohdsi.org/#/cohortdefinition/222
Prevalent obesity	https://atlas.ohdsi.org/#/cohortdefinition/224
Prevalent pre-existing condition of COVID risk factor	https://atlas.ohdsi.org/#/cohortdefinition/213
Prevalent tuberculosis	https://atlas.ohdsi.org/#/cohortdefinition/221
Prevalent tuberculosis broad	https://atlas.ohdsi.org/#/cohortdefinition/315
Prevalent Type 2 Diabetes Mellitus	https://atlas.ohdsi.org/#/cohortdefinition/311
Pulmonary Embolism events	https://atlas.ohdsi.org/#/cohortdefinition/274
Sepsis during hospitalization	https://atlas.ohdsi.org/#/cohortdefinition/277
Stillbirth	https://atlas.ohdsi.org/#/cohortdefinition/268
Stroke (ischemic or hemorrhagic) events	https://atlas.ohdsi.org/#/cohortdefinition/242
Suicide and suicidal ideation	https://atlas.ohdsi.org/#/cohortdefinition/235
Supraventricular arrythymia during hospitalization	https://atlas.ohdsi.org/#/cohortdefinition/270
Total cardiovascular disease events	https://atlas.ohdsi.org/#/cohortdefinition/246
tracheostomy during hospitalization	https://atlas.ohdsi.org/#/cohortdefinition/317
Transient ischemic attack events	https://atlas.ohdsi.org/#/cohortdefinition/243
Venous thromboembolic (pulmonary embolism and deep vein thrombosis) events	https://atlas.ohdsi.org/#/cohortdefinition/275
ventricular arrhythmia or cardiac arrest during hospitalization	https://atlas.ohdsi.org/#/cohortdefinition/269

Unraveling COVID-19: a large-scale characterization of 4.5 million COVID-19 cases using CHARYBDIS

Supplementary Figure 3. Standardized mean difference in conditions (A) and medication use (B) among patients diagnosed compared to patients hospitalized with COVID-19.

Individuals diagnosed compared with those hospitalized with COVID-19. Only databases with individuals in both cohorts and with at least 1 year of prior observation period were included. Conditions and medications from up to a year prior to the index date. Each dot represents one of these covariates with the colour indicating the type of condition/medication and the size of the dot reflecting the prevalence of the variable in the COVID-19 study populations.

Unraveling COVID-19: a large-scale characterization of 4.5 million COVID-19 cases using CHARYBDIS

A. Conditions by Database

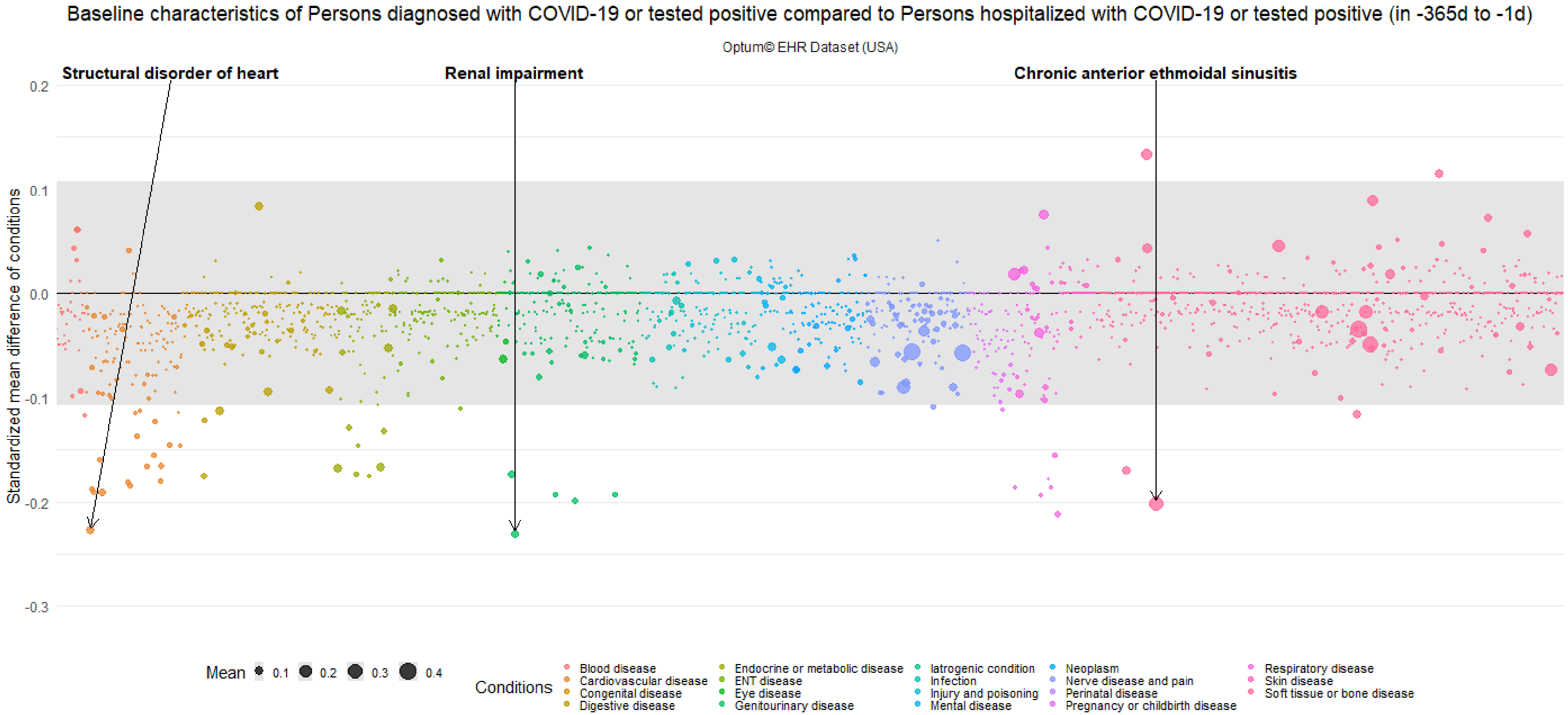

Unraveling COVID-19: a large-scale characterization of 4.5 million COVID-19 cases using CHARYBDIS

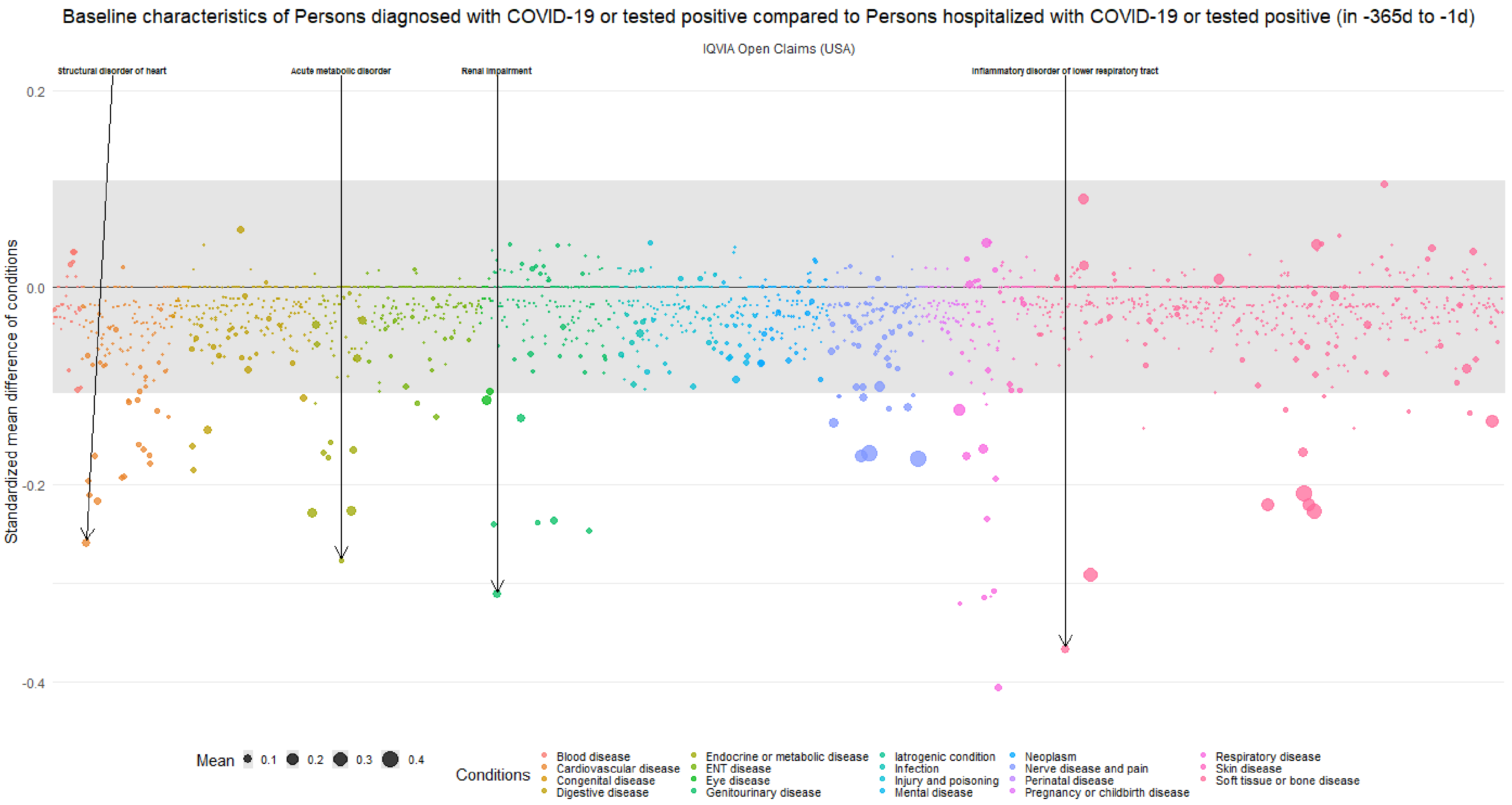

Unraveling COVID-19: a large-scale characterization of 4.5 million COVID-19 cases using CHARYBDIS

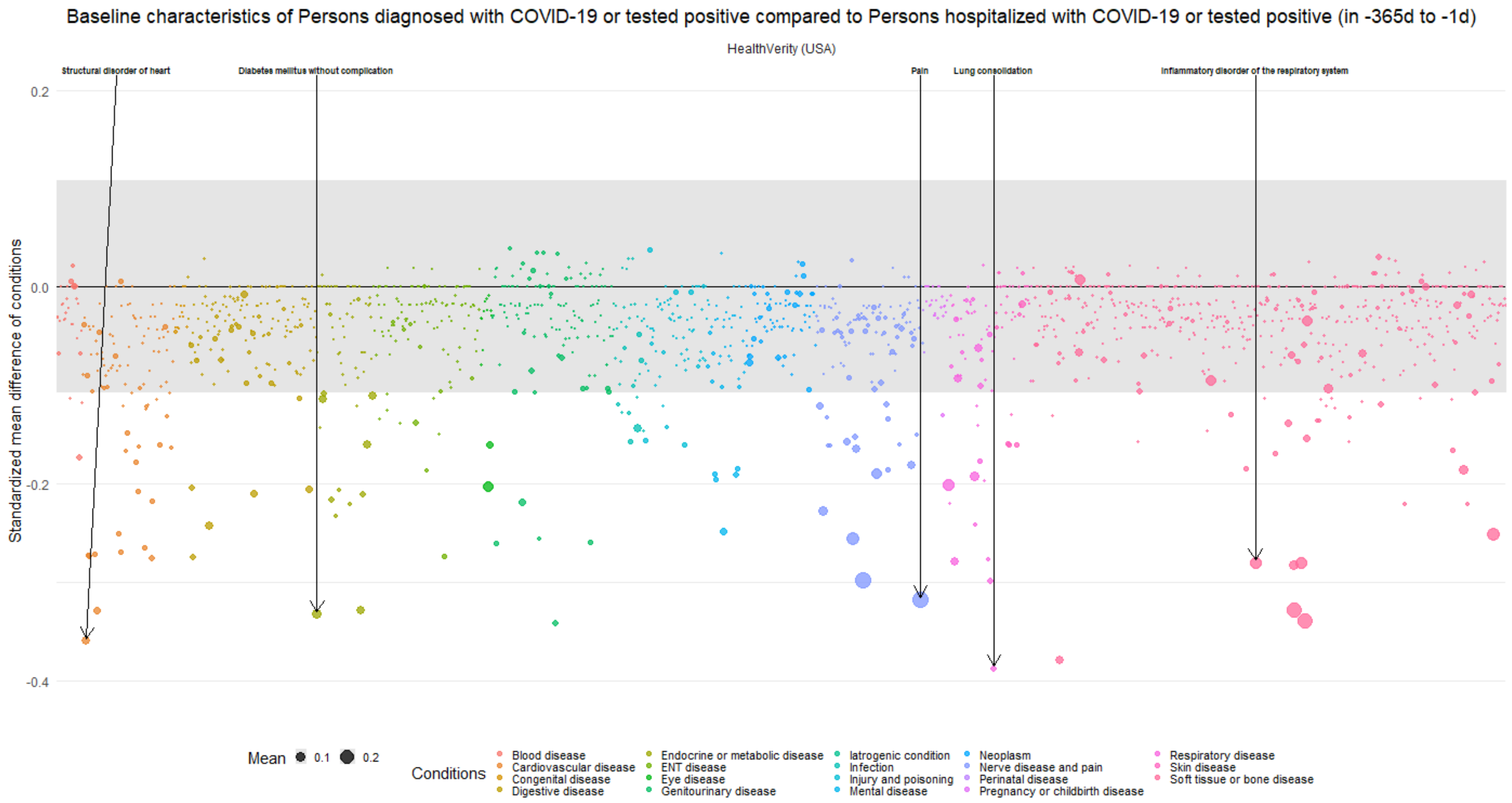

Unraveling COVID-19: a large-scale characterization of 4.5 million COVID-19 cases using CHARYBDIS

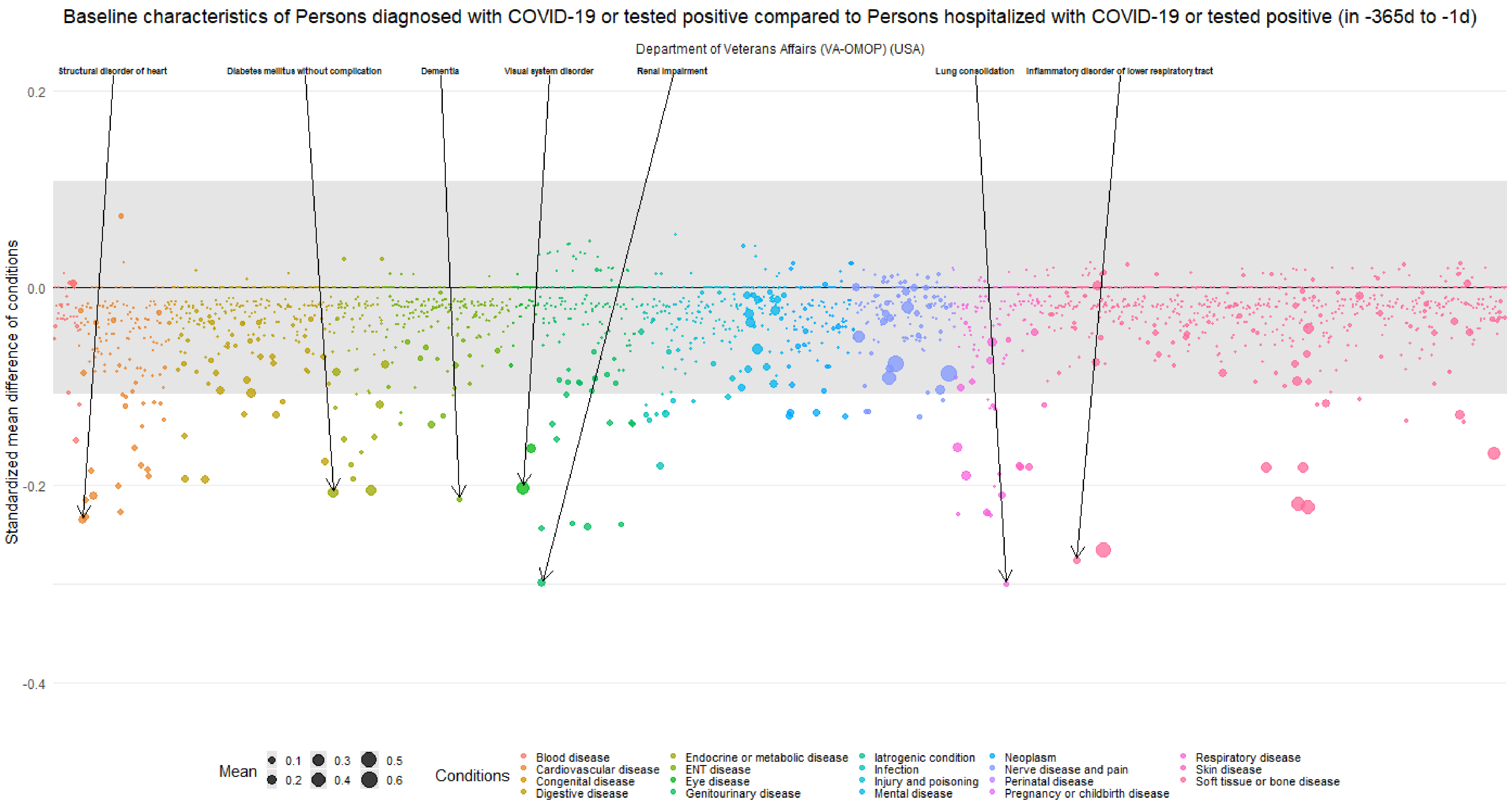

Unraveling COVID-19: a large-scale characterization of 4.5 million COVID-19 cases using CHARYBDIS

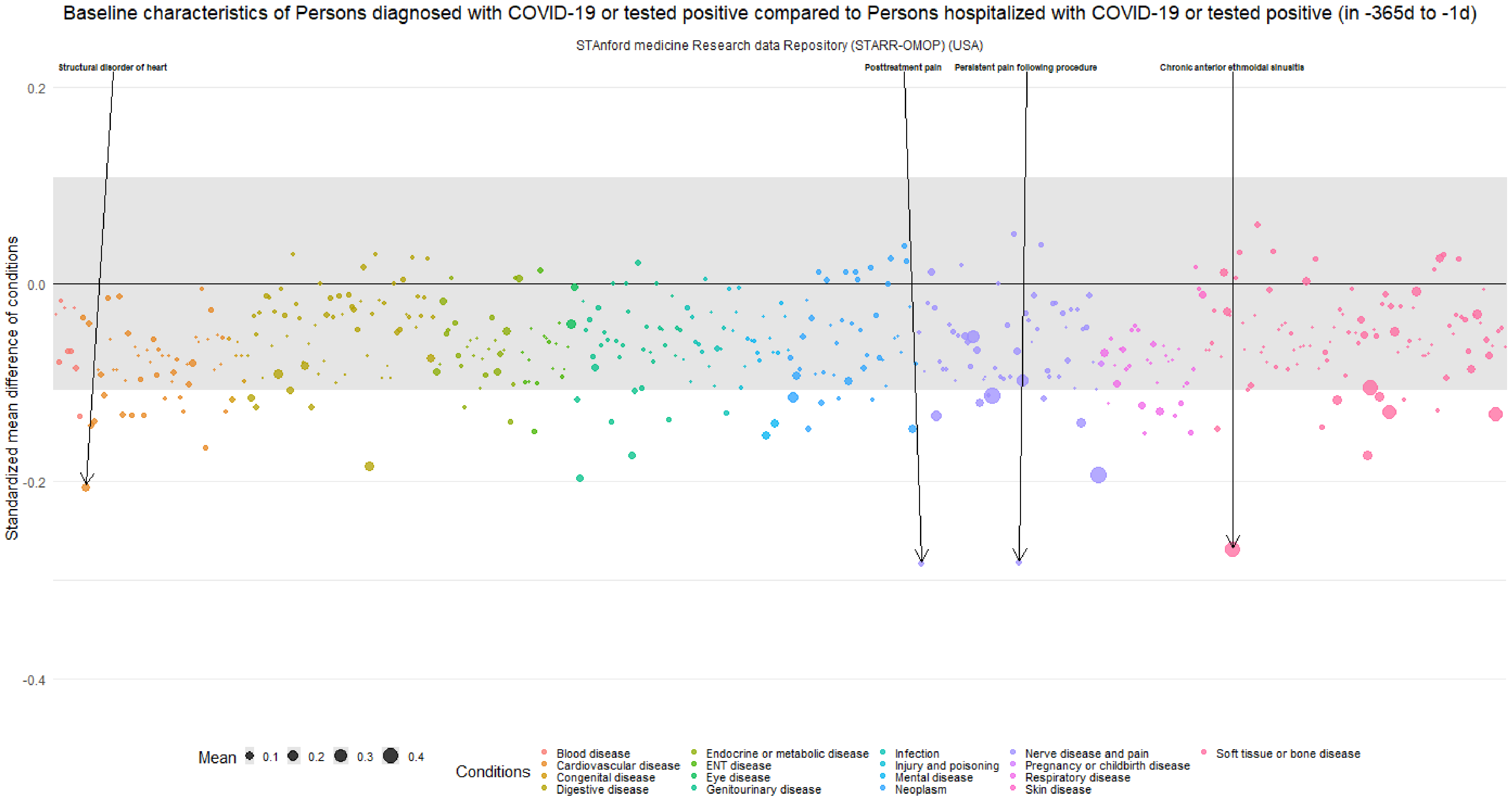

Unraveling COVID-19: a large-scale characterization of 4.5 million COVID-19 cases using CHARYBDIS

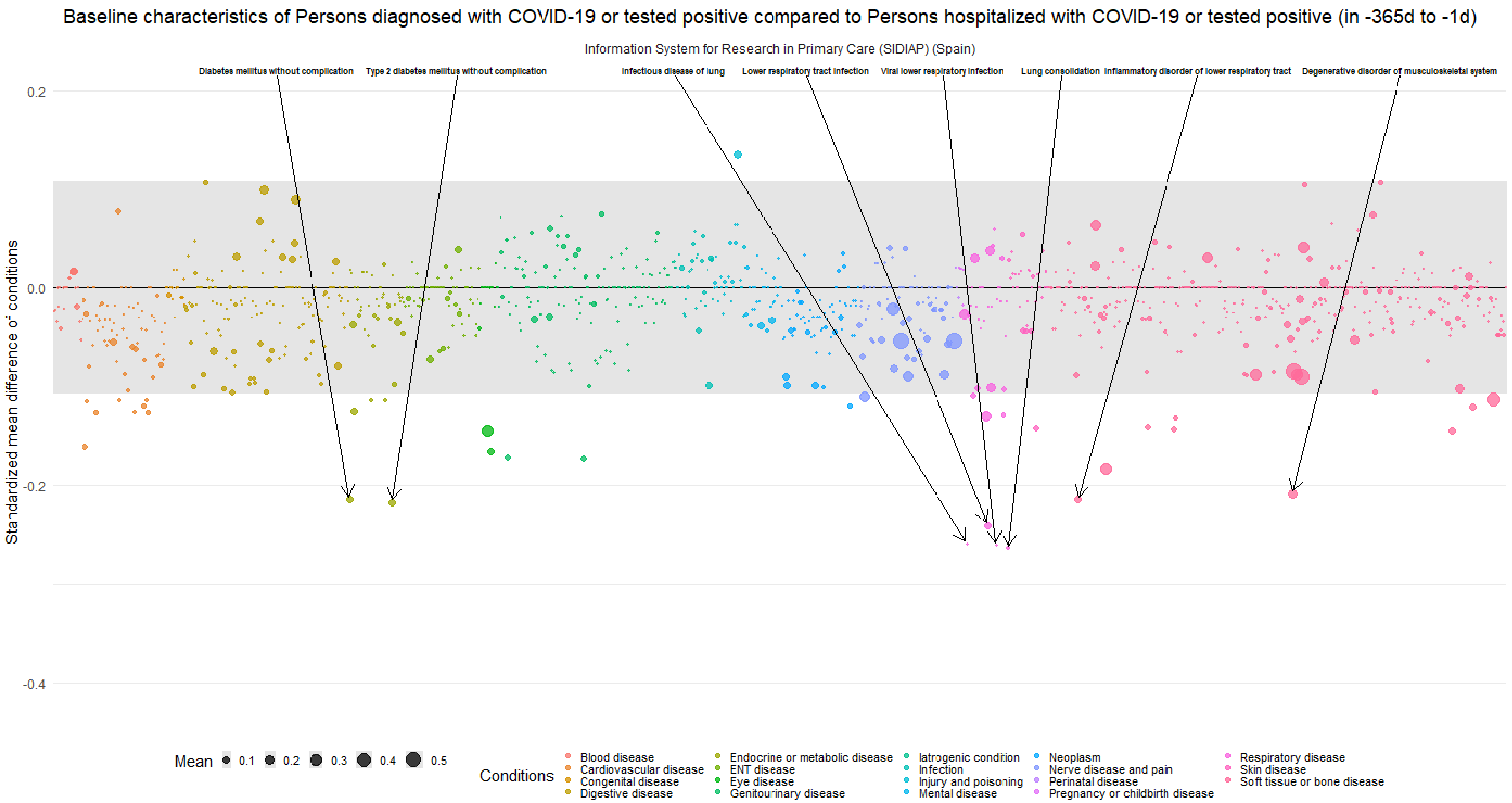

Unraveling COVID-19: a large-scale characterization of 4.5 million COVID-19 cases using CHARYBDIS

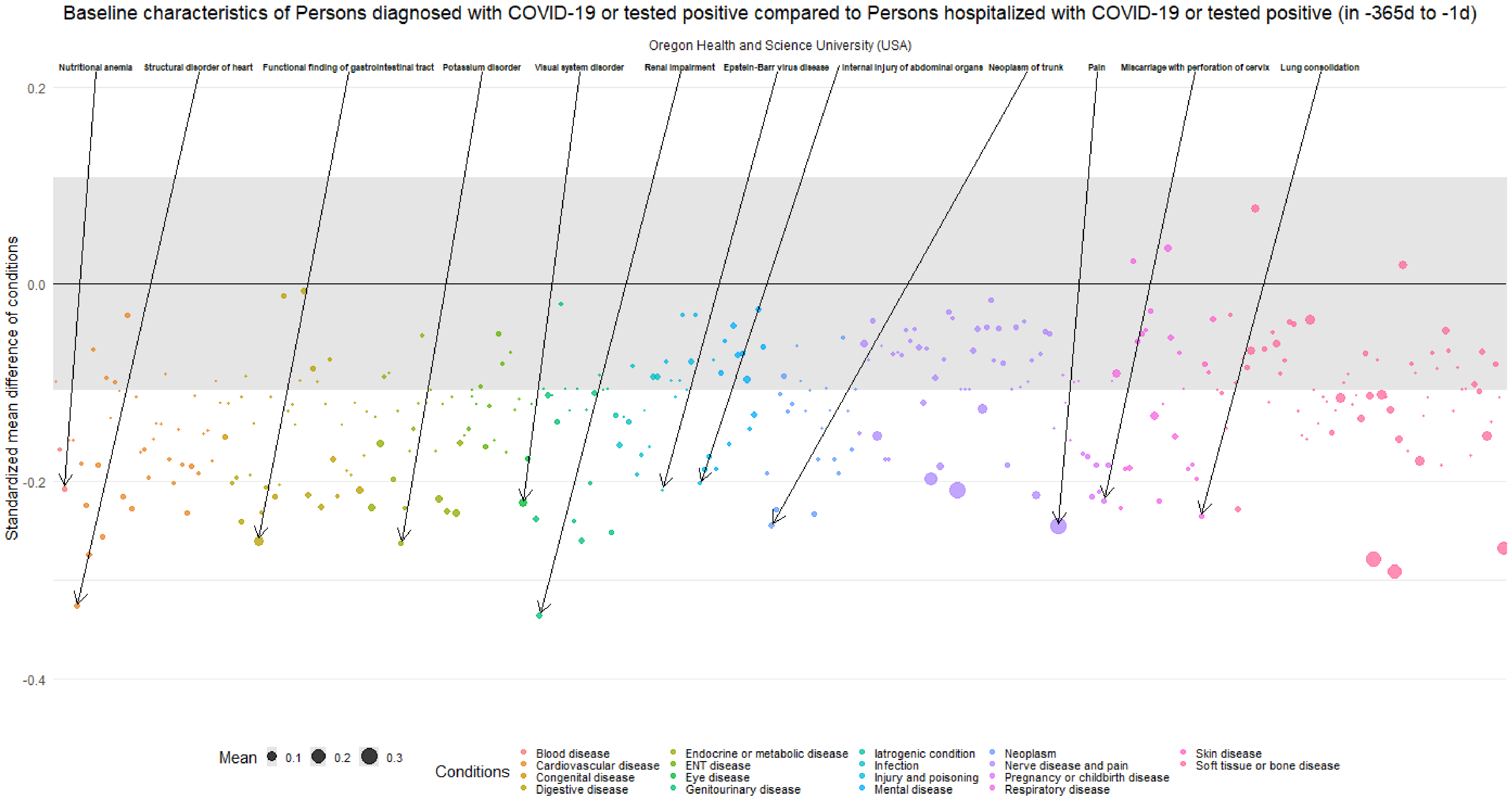

Unraveling COVID-19: a large-scale characterization of 4.5 million COVID-19 cases using CHARYBDIS

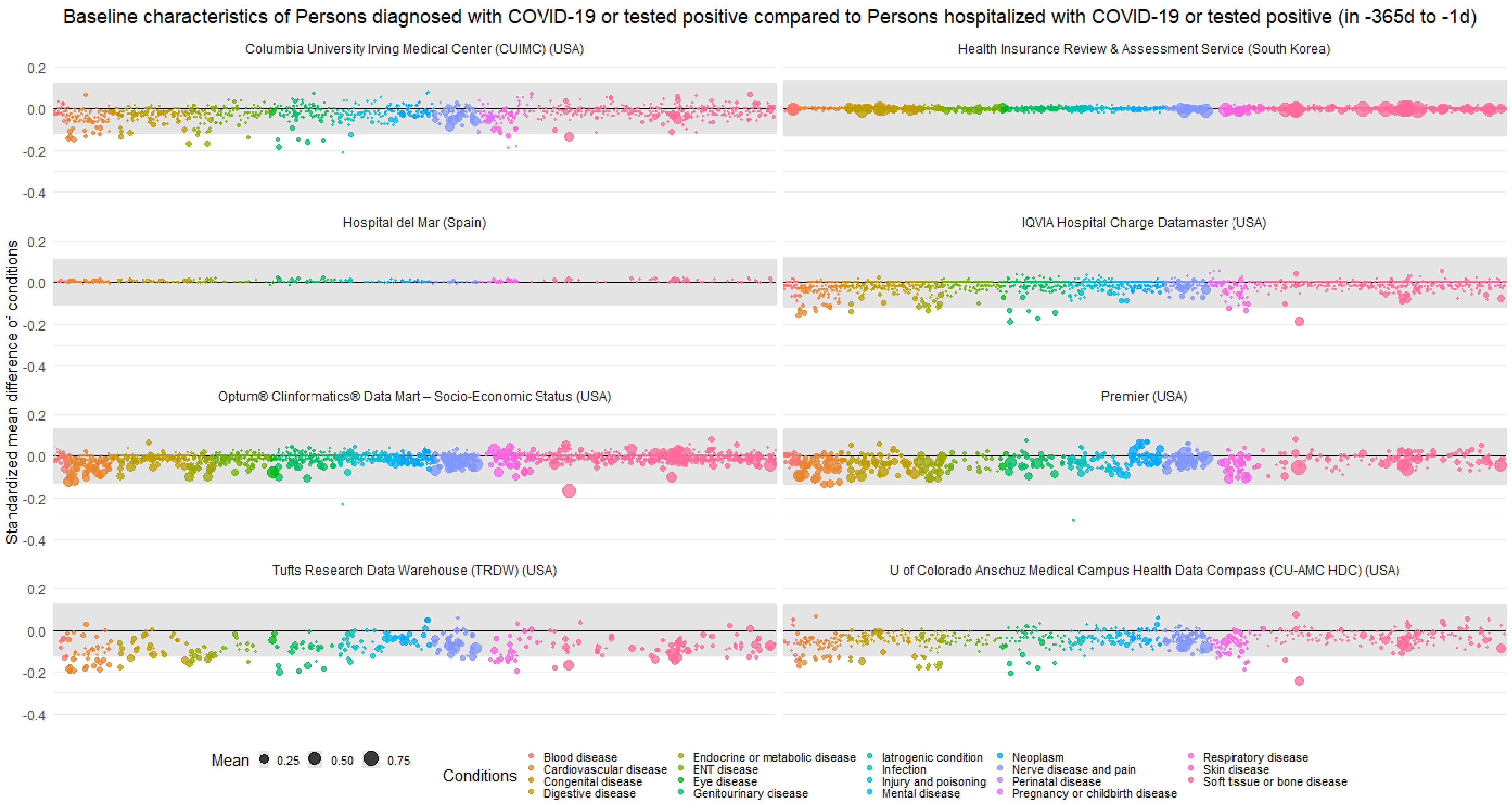

Unraveling COVID-19: a large-scale characterization of 4.5 million COVID-19 cases using CHARYBDIS

B. Medications

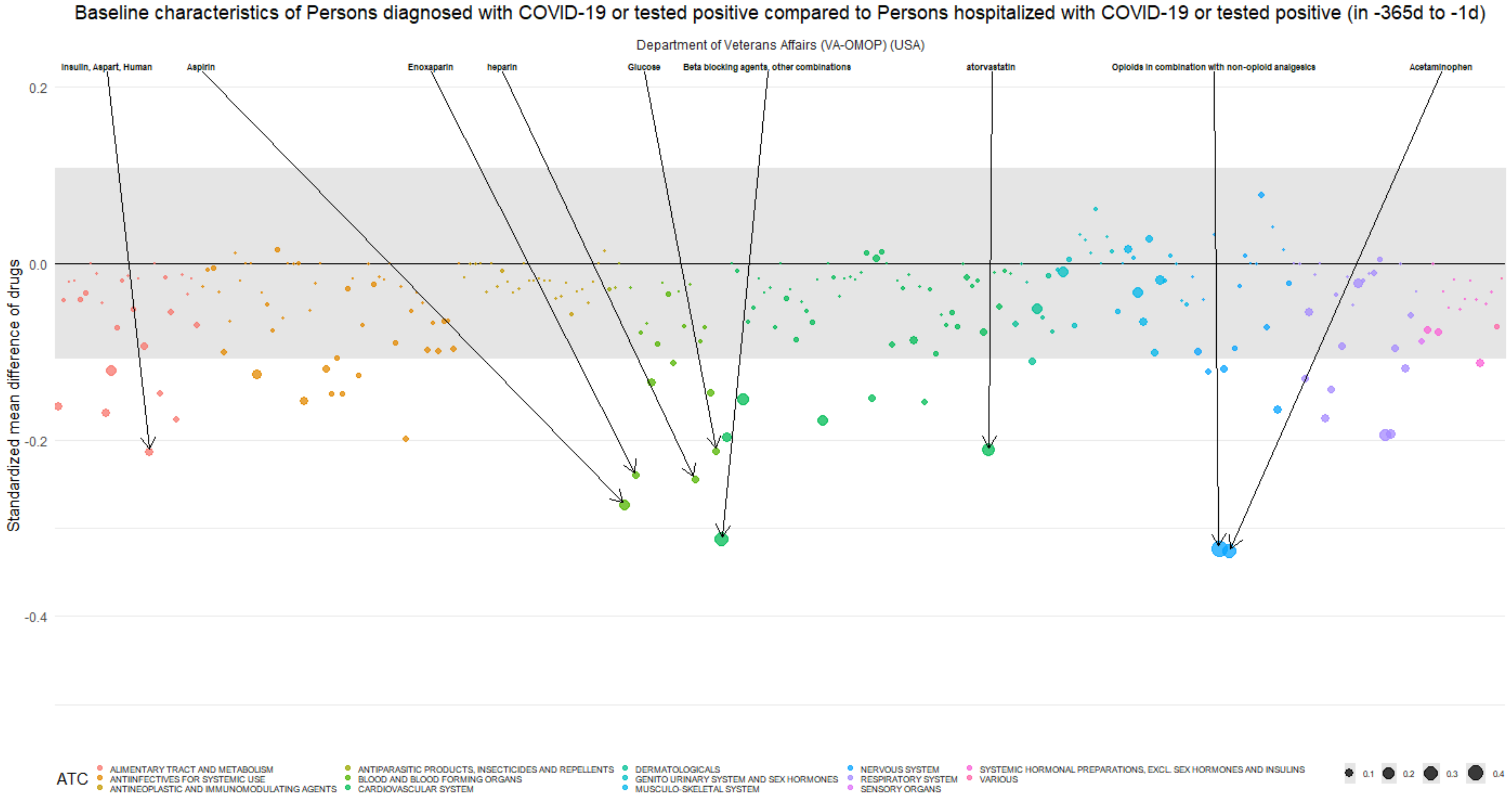

Unraveling COVID-19: a large-scale characterization of 4.5 million COVID-19 cases using CHARYBDIS

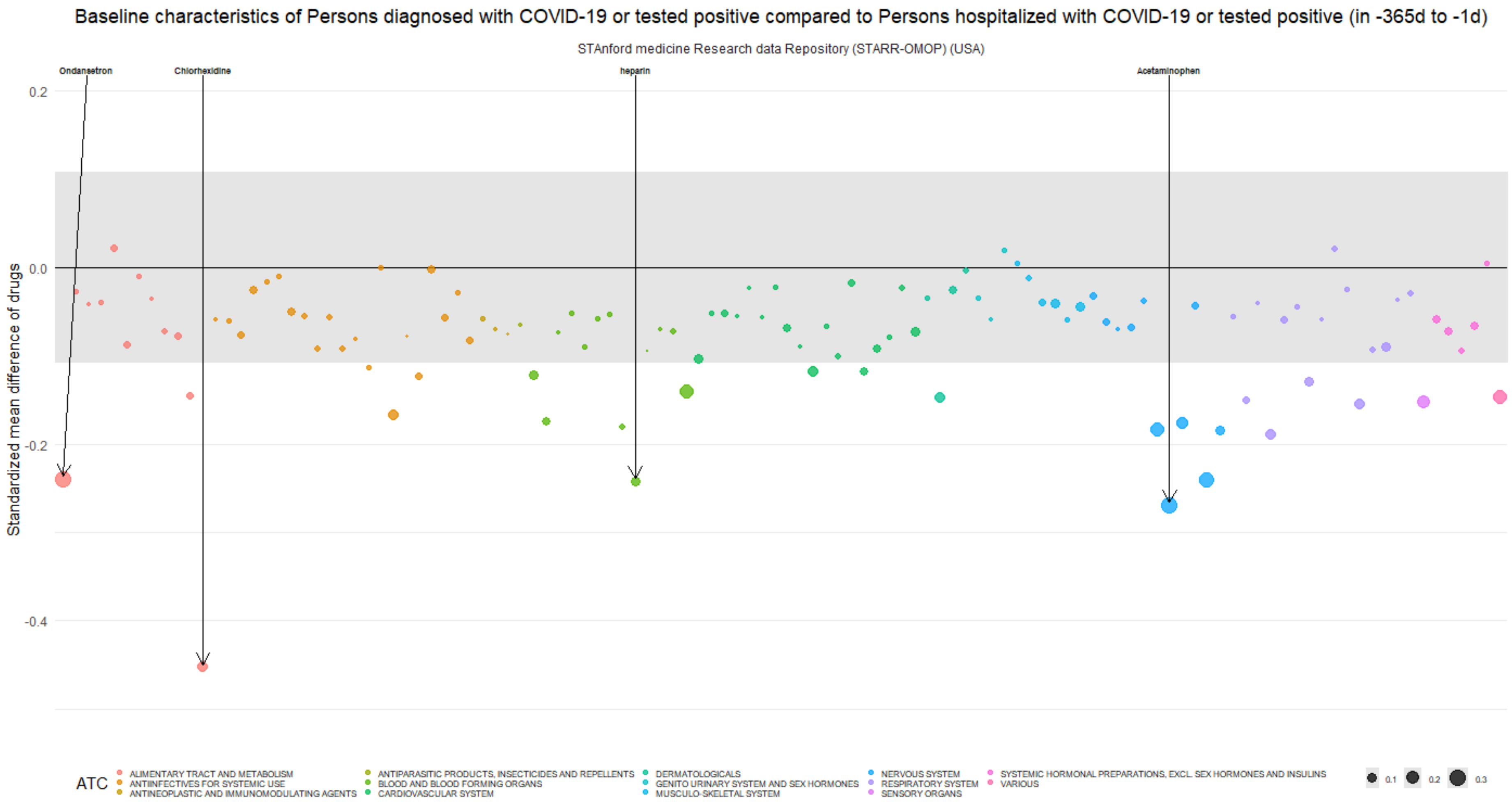

Unraveling COVID-19: a large-scale characterization of 4.5 million COVID-19 cases using CHARYBDIS

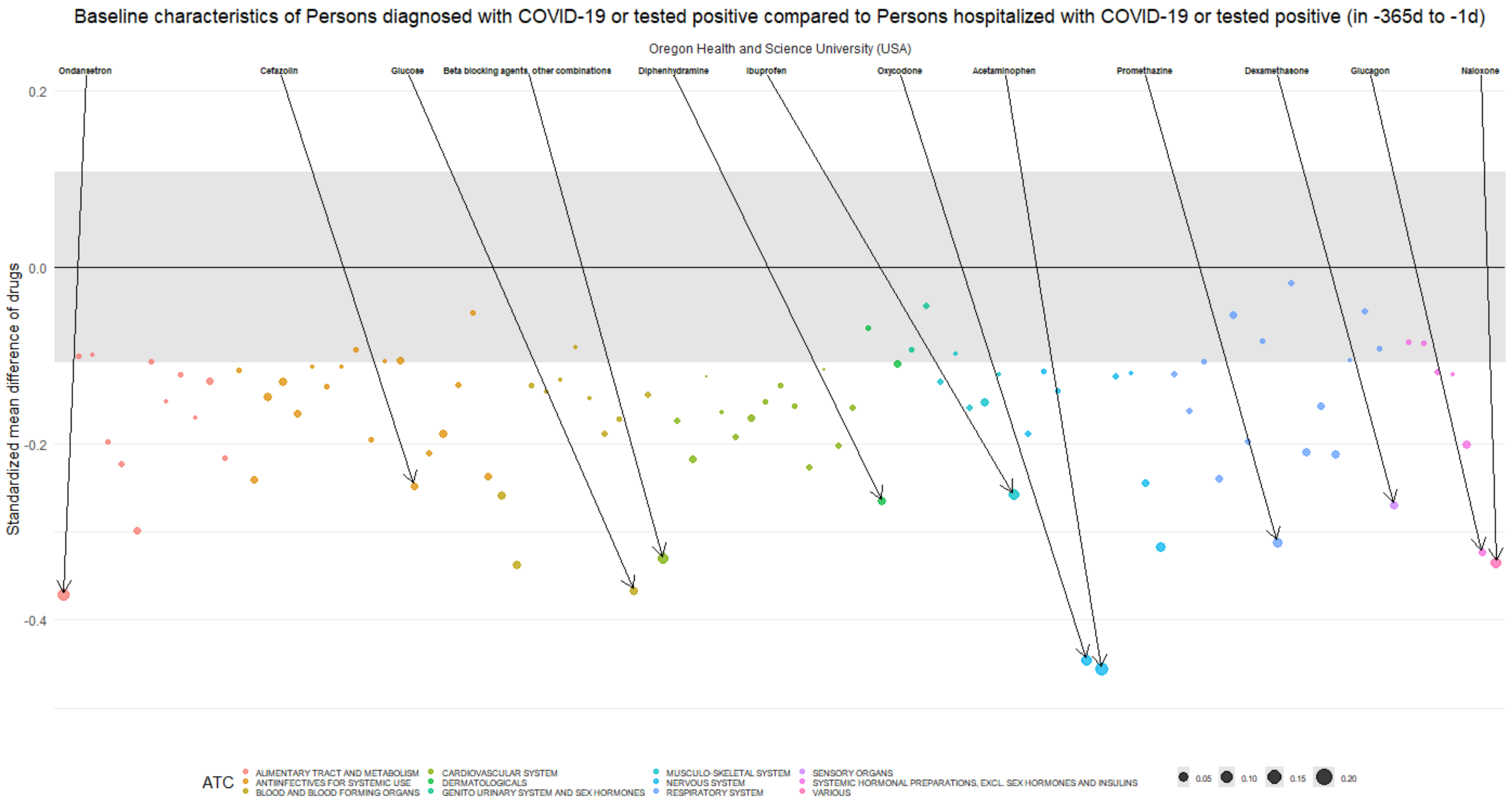

Unraveling COVID-19: a large-scale characterization of 4.5 million COVID-19 cases using CHARYBDIS

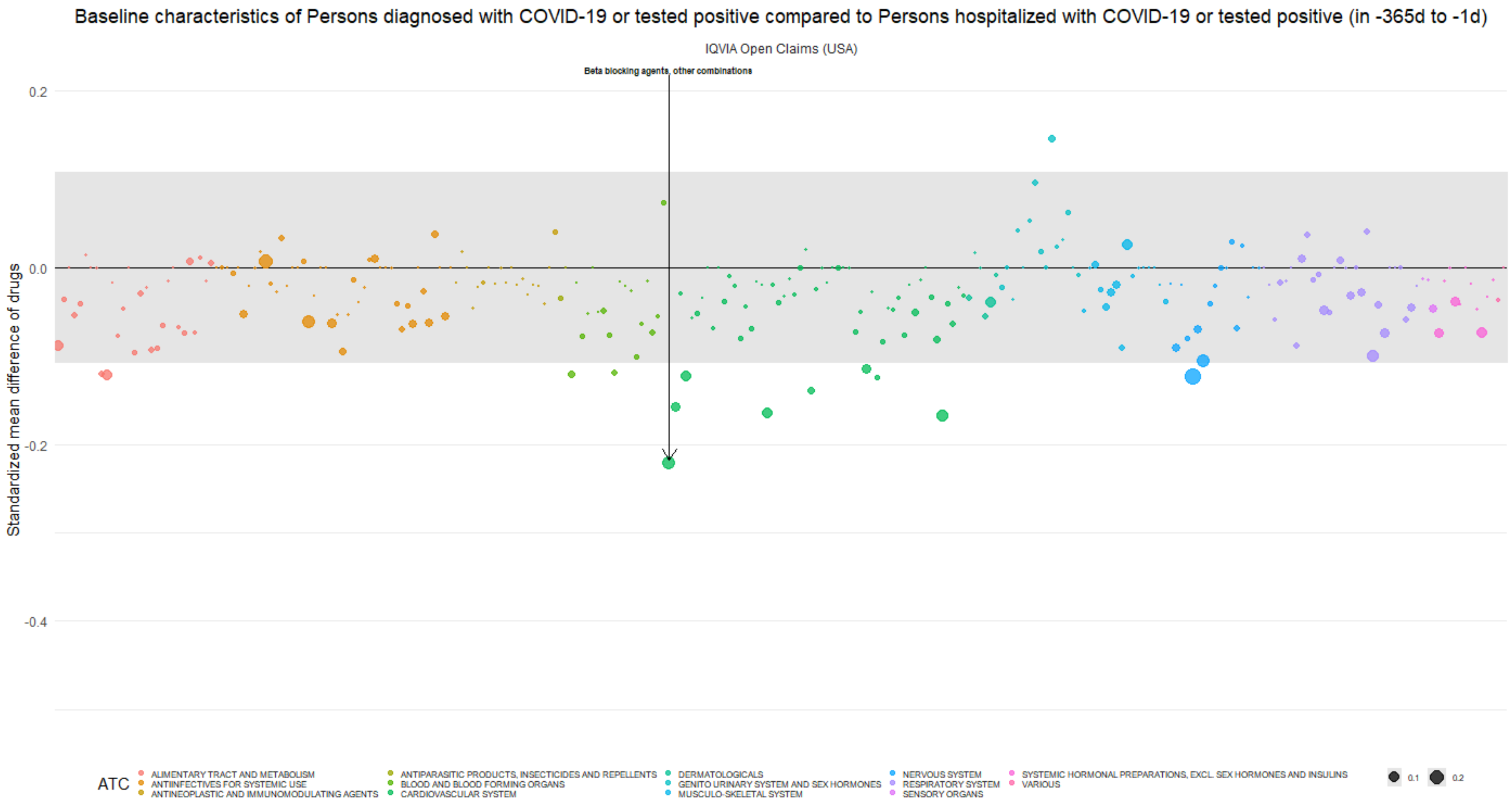

Unraveling COVID-19: a large-scale characterization of 4.5 million COVID-19 cases using CHARYBDIS

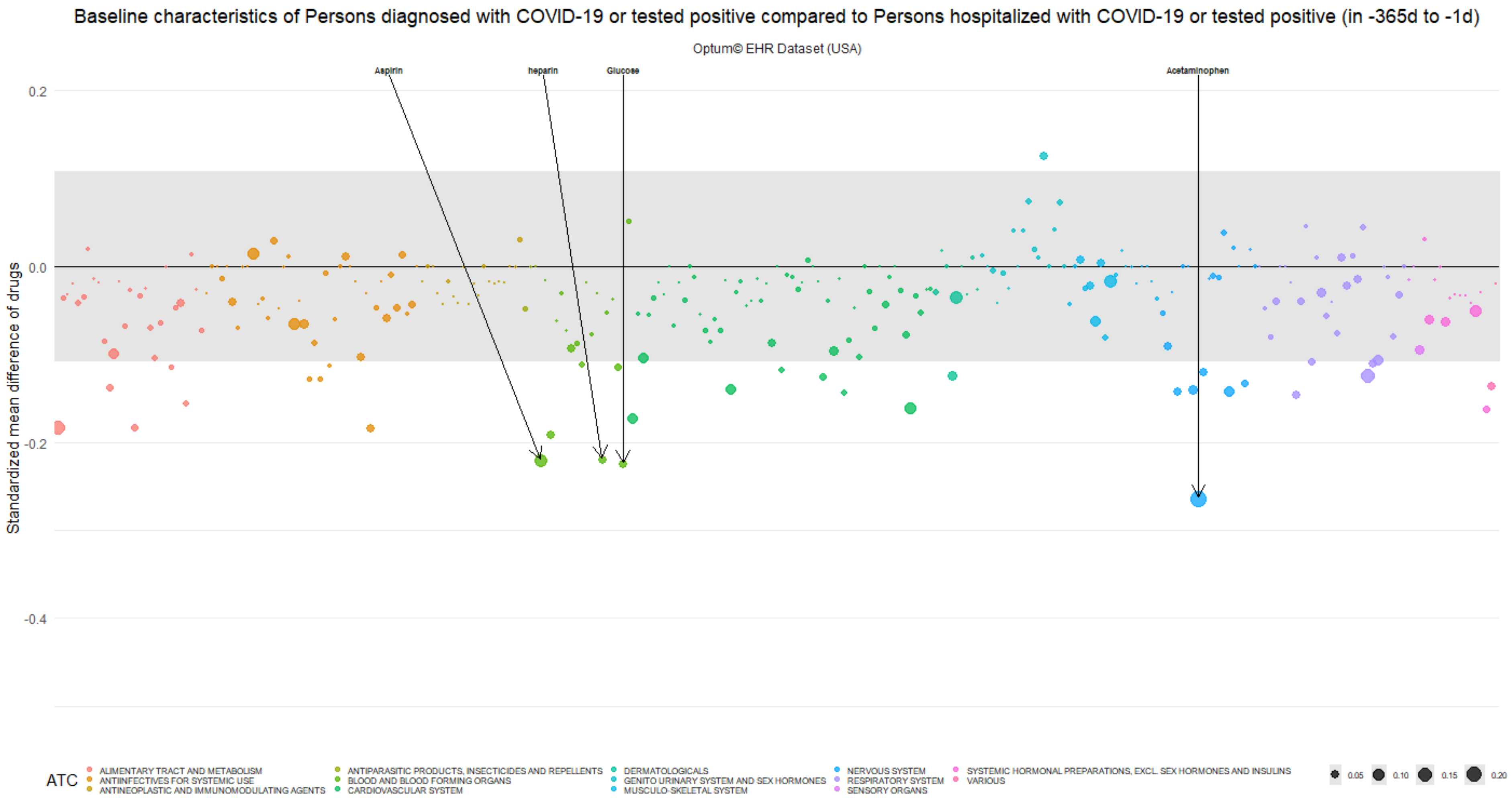

Unraveling COVID-19: a large-scale characterization of 4.5 million COVID-19 cases using CHARYBDIS

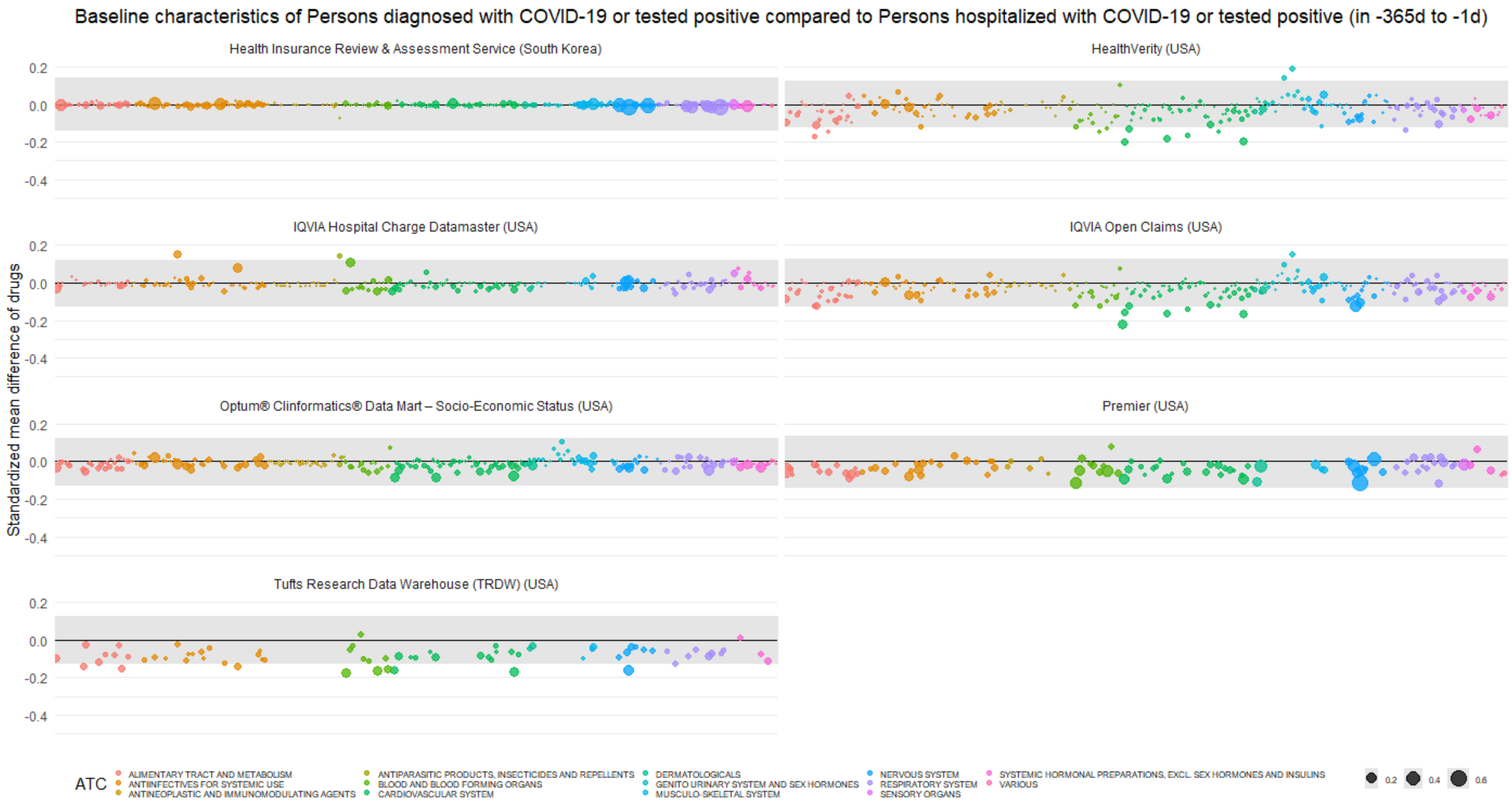

Unraveling COVID-19: a large-scale characterization of 4.5 million COVID-19 cases using CHARYBDIS

Supplementary Table 3. Number of persons in each CHARYBDIS cohort by database in the OHDSI COVID-19 Network.

Region	Database Name	No Prior Observation Period Required			At Least One Year of Prior Observation Period		
		Persons with a COVID-19 diagnosis or positive test	Persons hospitalized with a COVID-19	Persons hospitalized and requiring intensive services	Persons with a COVID-19 diagnosis or positive test	Persons hospitalized with a COVID-19	Persons hospitalized and requiring intensive services
**ASIA**							
	HIRA (South Korea)	NR	7,599	130	NR	7,599	130
	DCMC (South Korea)	559	46	NR	372	30	NR
	NFHCRD (China)	403	304	6	24	24	NR
**UNITED STATES**							
	HealthVerity (National)	587,683	22,887	1,289	114,173	7,581	471
	Premier (National)	417,650	156,187	36,735	530	259	84
	OptumEHR (National)	217,772	36,717	4,425	177,465	28,464	3,389
	Optum SES (National)	7,863	4,336	587	5,962	3,370	449
	STARR-OMOP (California)	4,788	744	62	3,328	615	46
	TRDW (Massachusetts)	1,250	326	102	664	186	40
	VA-OMOP (National)	57,937	10,951	1,904	55,557	10,471	1,774
	IQVIA-OpenClaims (National)	2,785,812	533,997	47,715	2,578,663	502,933	45,549
	CUIMC (New York)	37,773	7,353	135	32,090	5,707	98
	CU-AMC-HDC (Colorado)	9,481	1,874	452	7,270	1,434	344
	UWM-CRD (Washington)	3,245	733	117	1,848	534	68
	IQVIA Hospital Charge Datamaster (National)	209,534	77,853	18,274	128,140	46,865	10,971
	OHSU (Oregon)	11,187	627	69	5,618	466	42
**EUROPE**							
	HM-Hospitales (Spain)	NR	2,544	1,397	NR	NR	NR
	SIDIAP (Spain)	124,305	18,369	NR	122,141	18,202	NR
	SIDIAP-H (Spain)	43,411	7,197	NR	42,786	7,139	NR
	HMAR (Spain)	NR	2,686	228	NR	2,011	181
	IPCI (Netherlands)	3,306	60	NR	3,047	59	NR
	CPRD (UK)	3,864	NR	NR	3,372	NR	NR
	IQVIA-LPD France	23,592	NR	NR	17,18	NR	NR
	IQVIA-DA Germany	11,500	NR	NR	7,971	NR	NR
	IQVIA-LPD Italy	4,816	NR	NR	4,488	NR	NR
**TOTAL**		**4,567,731**	**893,390**	**113,627**	**3,295,509**	**643,949**	**63,636**

* Databases with no prior observation period available: NFHCRD, HM-Hospitales.

Abbreviations: Clinical Practice Research Datalink (CPRD), Colorado University Anschuz Medical Campus Health Data Compass (CU-AMC HDC), Columbia University Irving Medical Center (CUIMC), Daegu Catholic University Medical Center (DCMC) Health Insurance Review & Assessment Service (HIRA), Hospital del Mar (HMAR), Integrated Primary Care Information (IPCI), IQVIA Disease Analyser Germany (DA Germany), IQVIA Longitudinal Patient Data France (LPD France), IQVIA Longitudinal Patient Data Italy (LPD Italy), Nanfang Hospital COVID-19 Research Database (NFHCRD) Oregon Health & Science University (OHSU) Information System for Research in Primary Care (SIDIAP), Information System for Research in Primary Care – Hospitalization Linked Data (SIDIAP-H), STAnford medicine Research data Repository (STARR-OMOP), Tufts MC Research Data Warehouse (TRDW), UW Medicine COVID Research Dataset (UWM-CRD) Department of Veterans Affairs (VA-OMOP), NR: Not Reported by Data Partner.

Supplementary Table 4. Distributions of persons diagnosed with a COVID-19 diagnosis or SARS-CoV-2 positive test in each cohort (1 year observation period required)

	ASIA	UNITED STATES													EUROPE					
	DCMC	HealthVerity	Premier	OPTUM-EHR	OPTUM-SES	STARR-OMOP	TRDW	VA-OMOP	IQVIA OpenClaims	IQVIA Hospital CDM	CUIMC	CU-AMC-HDC	UWM-CRD	OHSU	SIDIAP	IPCI	CPRD	IQVIA LPD France	IQVIA DA Germany	IQVIA LPD Italy
COVID-19 Cases (N)	372	114,173	194	129,512	5,962	3,328	664	55,557	2,578,663	94,106	8,519	7,270	1,848	5,618	122,141	3,047	3,372	17,180	7,971	4,488
N tested	NR	620,962	1,289	835,797	29,506	39,877	3,719	501,106	6,151,004	464,495	18,053	99,112	53,581	60,588	171,81	NR	4,908	NR	NR	NR
Tested positive, n (%)	NR	82,917 (72.6)	NR	57,381 (44.3)	NR	902 (27.1)	520 (78.3)	31,293 (56.3)	NR	NR	5,625 (66.0)	NR	1,777 (96.2)	3,911 (69.6)	38,657 (31.6)	NR	1,813 (53.8)	NR	NR	NR
**Follow up, n (%)**																				
Full 30-day follow up	159 (42.7)	27,286 (23.9)	59 (30.4)	71,267 (55.0)	915 (15.3)	2,352 (70.7)	460 (69.3)	43,575 (78.4)	1,818,389 (70.5)	15,686 (16.7)	1,680 (19.7)	6,758 (93.0)	979 (53.0)	2,733 (48.6)	80,532 (65.9)	2,417 (79.3)	2,403 (71.3)	8,047 (46.8)	4,792 (60.1)	3,358 (74.8)
< 30-day follow up	213 (57.3)	86,887 (76.1)	135 (69.6)	58,245 (45.0)	5,047 (84.7)	976 (29.3)	204 (30.7)	11,978 (21.6)	760,274 (29.5)	78,420 (83.3)	6,839 (80.3)	499 (6.9)	869 (47.0)	2,885 (51.4)	41,609 (34.1)	630 (20.7)	969 (28.7)	9,133 (53.2)	3,179 (39.9)	1,130 (25.2)
**Conditions, n (%)**																				
Type 2 Diabetes Mellitus	105 (28.2)	14,900 (13.1)	85 (43.8)	24,248 (18.7)	2,309 (38.7)	519 (15.6)	167 (25.2)	18,939 (34.1)	715,513 (27.7)	26,859 (28.5)	1,903 (22.3)	1,240 (17.1)	322 (17.4)	562 (10.0)	9,916 (8.1)	483 (15.9)	515 (15.3)	1,212 (7.1)	1,027 (12.9)	437 (9.7)
Hypertension	150 (40.3)	25,405 (22.3)	156 (80.4)	50,160 (38.7)	3,708 (62.2)	1,246 (37.4)	286 (43.1)	34,093 (61.4)	1,245,436 (48.3)	46,110 (49.0)	3,672 (43.1)	2,461 (33.9)	624 (33.8)	1,000 (17.8)	21,289 (17.4)	676 (22.2)	756 (22.4)	3,260 (19.0)	2,418 (30.3)	1,618 (36.1)
Heart disease	102 (27.4)	14,469 (12.7)	122 (62.9)	36,362 (28.1)	3,199 (53.7)	923 (27.7)	220 (33.1)	24,542 (44.2)	926,008 (35.9)	26,462 (28.1)	3,068 (36.0)	1,734 (23.9)	372 (20.1)	716 (12.7)	17,724 (14.5)	460 (15.1)	695 (20.6)	1,152 (6.7)	1,864 (23.4)	993 (22.1)
Prior cancer	28 (7.5)	4,857 (4.3)	74 (38.1)	17,630 (13.6)	1,321 (22.2)	821 (24.7)	100 (15.1)	10,760 (19.4)	315,523 (12.2)	9,262 (9.8)	1,433 (16.8)	806 (11.1)	153 (8.3)	419 (7.5)	8,854 (7.2)	257 (8.4)	285 (8.5)	658 (3.8)	619 (7.8)	528 (11.8)
Hepatitis C	NR	506 (0.4)	9 (4.6)	1,307 (1.0)	87 (1.5)	60 (1.8)	35 (5.3)	3,065 (5.5)	39,822 (1.5)	1,693 (1.8)	140 (1.6)	88 (1.2)	52 (2.8)	82 (1.5)	645 (0.5)	NR	NR	40 (0.2)	31 (0.4)	52 (1.2)
Obesity	27 (7.3)	12,249 (10.7)	69 (35.6)	65,750 (50.8)	2,145 (36.0)	1,157 (34.8)	283 (42.6)	25,030 (45.1)	734,604 (28.5)	23,941 (25.4)	3,446 (40.5)	2,920 (40.2)	198 (10.7)	886 (15.8)	36,439 (29.8)	616 (20.2)	1,379 (40.9)	2,073 (12.1)	1,277 (16.0)	662 (14.8)

Dementia	6 (1.6)	2,900 (2.5)	27 (13.9)	4,754 (3.7)	755 (12.7)	36 (1.1)	22 (3.3)	3,977 (7.2)	216,248 (8.4)	5,257 (5.6)	473 (5.6)	212 (2.9)	103 (5.6)	89 (1.6)	6,003 (4.9)	57 (1.9)	300 (8.9)	50 (0.3)	285 (3.6)	74 (1.6)
Autoimmune condition	48 (12.9)	5,884 (5.2)	28 (14.4)	12,897 (10.0)	1,334 (22.4)	406 (12.2)	130 (19.6)	10,087 (18.2)	431,640 (16.7)	7,856 (8.3)	1,363 (16.0)	696 (9.6)	128 (6.9)	388 (6.9)	8,238 (6.7)	472 (15.5)	382 (11.3)	1,398 (8.1)	1,139 (14.3)	626 (13.9)
COPD without asthma	NR	6,500 (5.7)	50 (25.8)	11,056 (8.5)	1,226 (20.6)	218 (6.6)	78 (11.7)	12,610 (22.7)	294,741 (11.4)	9,566 (10.2)	770 (9.0)	692 (9.5)	99 (5.4)	226 (4.0)	15,803 (12.9)	200 (6.6)	261 (7.7)	648 (3.8)	816 (10.2)	340 (7.6)
Asthma without COPD	16 (4.3)	8,477 (7.4)	25 (12.9)	20,049 (15.5)	955 (16.0)	501 (15.1)	104 (15.7)	6,256 (11.3)	435,591 (16.9)	10,476 (11.1)	1,338 (15.7)	1,035 (14.2)	159 (8.6)	533 (9.5)	7,542 (6.2)	311 (10.2)	488 (14.5)	2,157 (12.6)	1,013 (12.7)	413 (9.2)
Pregnant women	NR	913 (0.8)	NR	3,191 (2.5)	82 (1.4)	31 (0.9)	13 (2.0)	83 (0.1)	36,955 (1.4)	1,850 (2.0)	317 (3.7)	168 (2.3)	28 (1.5)	110 (2.0)	660 (0.5)	28 (0.9)	8 (0.2)	168 (1.0)	30 (0.4)	61 (1.4)
Flu-like symptom episodes	38 (10.2)	26,051 (22.8)	85 (43.8)	39,731 (30.7)	4,167 (69.9)	625 (18.8)	337 (50.8)	11,450 (20.6)	729,102 (28.3)	29,535 (31.4)	2,514 (29.5)	2,590 (35.6)	689 (37.3)	2,104 (37.5)	25,159 (20.6)	339 (11.1)	120 (3.6)	2,995 (17.4)	437 (5.5)	120 (2.7)
CKD broad	151 (40.6)	5,714 (5.0)	86 (44.3)	16,497 (12.7)	1,608 (27.0)	379 (11.4)	NR	10,194 (18.3)	361,527 (14.0)	12,934 (13.7)	1,143 (13.4)	684 (9.4)	182 (9.8)	260 (4.6)	8,135 (6.7)	193 (6.3)	463 (13.7)	187 (1.1)	528 (6.6)	185 (4.1)
ESRD	150 (40.3)	1,363 (1.2)	23 (11.9)	2,811 (2.2)	332 (5.6)	119 (3.6)	NR	3,259 (5.9)	95,502 (3.7)	4,412 (4.7)	586 (6.9)	160 (2.2)	45 (2.4)	51 (0.9)	8 (0.0)	NR	15 (0.4)	NR	27 (0.3)	NR
HIV/AIDS	NR	546 (0.5)	NR	685 (0.5)	52 (0.9)	20 (0.6)	NR	805 (1.4)	24,401 (0.9)	1,078 (1.1)	154 (1.8)	53 (0.7)	39 (2.1)	39 (0.7)	288 (0.2)	NR	NR	78 (0.5)	15 (0.2)	19 (0.4)

Abbreviations: Clinical Practice Research Datalink (CPRD), Colorado University Anschuz Medical Campus Health Data Compass (CU-AMC HDC), Columbia University Irving Medical Center (CUIMC), Daegu Catholic University Medical Center (DCMC) Health Insurance Review & Assessment Service (HIRA), Hospital del Mar (HMAR), Integrated Primary Care Information (IPCI), IQVIA Disease Analyser Germany (DA Germany), IQVIA Longitudinal Patient Data France (LPD France), IQVIA Longitudinal Patient Data Italy (LPD Italy), Nanfang Hospital COVID-19 Research Database (NFHCRD) Oregon Health & Science University (OHSU) Information System for Research in Primary Care (SIDIAP), Information System for Research in Primary Care – Hospitalization Linked Data (SIDIAP-H), STAnford medicine Research data Repository (STARR-OMOP), Tufts MC Research Data Warehouse (TRDW), UW Medicine COVID Research Dataset (UWM-CRD) Department of Veterans Affairs (VA-OMOP), NR: Not Reported by Data Partner. COPD: Chronic obstructive pulmonary disease; CKD: Chronic kidney disease; ESRD: End-stage renal disease; HIV/AIDS: Human immunovirus deficiency/Acquired immunodeficiency syndrome.

Supplementary Table 5. Distributions of persons hospitalized with COVID-19 diagnosis or SARS-CoV-2 positive test in each cohort (1 year observation period required)

	ASIA	UNITED STATES												EUROPE	
	HIRA	HealthVerity	OPTUM-EHR	OPTUM-SES	STARR-OMOP	TRDW	VA-OMOP	IQVIA OpenClaims	IQVIA Hospital CDM	CUIMC	CU-AMC-HDC	UWM-CRD	OHSU	SIDIAP	HMAR
**N**	7,599	7,581	22,024	3,370	615	186	10,471	502,933	33,954	2,600	1,434	534	466	18,202	2,011
**Hospitalized with a positive test, n (%)**	NR	4,221 (55.7)	10,304 (46.8)	NR	86 (14.0)	140 (75.3)	8,352 (79.8)	NR	NR	2,344 (90.2)	NR	494 (92.5)	248 (53.2)	13,561 (74.5)	609 (30.3)
**Follow up, n (%)**															

Full 30-day follow up	7,359 (96.8)	4,294 (56.6)	14,744 (66.9)	627 (18.6)	556 (90.4)	NR	8,353 (79.8)	398,650 (79.3)	8,316 (24.5)	735 (28.3)	1,385 (96.6)	319 (59.7)	365 (78.3)	12,170 (66.9)	1,040 (51.7)
< 30-day follow up	240 (3.2)	3,287 (43.4)	7,280 (33.1)	2,743 (81.4)	59 (9.6)	NR	2,115 (20.2)	104,283 (20.7)	25,638 (75.5)	1,865 (71.7)	49 (3.4)	215 (40.3)	101 (21.7)	6,032 (33.1)	971 (48.3)
**Conditions, n (%)**															
Type 2 Diabetes Mellitus	1,760 (23.2)	2,879 (38.0)	7,977 (36.2)	1,581 (46.9)	138 (22.4)	77 (41.4)	5,773 (55.1)	250,468 (49.8)	13,990 (41.2)	1,065 (41.0)	556 (38.8)	174 (32.6)	150 (32.2)	3,286 (18.1)	268 (13.3)
Hypertension	1,943 (25.6)	4,512 (59.5)	13,993 (63.5)	2,498 (74.1)	342 (55.6)	116 (62.4)	8,996 (85.9)	384,508 (76.5)	22,219 (65.4)	1,708 (65.7)	904 (63.0)	324 (60.7)	246 (52.8)	5,636 (31.0)	594 (29.5)
Heart disease	1,271 (16.7)	3,414 (45.0)	11,300 (51.3)	2,238 (66.4)	264 (42.9)	100 (53.8)	7,339 (70.1)	314,973 (62.6)	13,803 (40.7)	1,418 (54.5)	688 (48.0)	233 (43.6)	215 (46.1)	5,142 (28.2)	335 (16.7)
Prior history of cancer	410 (5.4)	797 (10.5)	4,451 (20.2)	939 (27.9)	244 (39.7)	46 (24.7)	3,383 (32.3)	105,931 (21.1)	4,706 (13.9)	561 (21.6)	265 (18.5)	75 (14.0)	127 (27.3)	2,610 (14.3)	169 (8.4)
Hepatitis C	61 (0.8)	99 (1.3)	418 (1.9)	59 (1.8)	15 (2.4)	13 (7.0)	1,032 (9.9)	14,294 (2.8)	944 (2.8)	78 (3.0)	35 (2.4)	25 (4.7)	33 (7.1)	133 (0.7)	35 (1.7)
Obesity	16 (0.2)	1,633 (21.5)	13,279 (60.3)	1,397 (41.5)	274 (44.6)	94 (50.5)	5,656 (54.0)	189,307 (37.6)	9,651 (28.4)	1,408 (54.2)	830 (57.9)	109 (20.4)	136 (29.2)	8,405 (46.2)	251 (12.5)
Dementia	436 (5.7)	1,087 (14.3)	2,039 (9.3)	563 (16.7)	16 (2.6)	17 (9.1)	2,055 (19.6)	80,728 (16.1)	3,155 (9.3)	364 (14.0)	123 (8.6)	84 (15.7)	22 (4.7)	1,099 (6.0)	71 (3.5)
Autoimmune condition	813 (10.7)	946 (12.5)	3,112 (14.1)	842 (25.0)	85 (13.8)	54 (29.0)	3,152 (30.1)	136,193 (27.1)	3,807 (11.2)	557 (21.4)	209 (14.6)	61 (11.4)	72 (15.5)	1,702 (9.4)	81 (4.0)
COPD without asthma	145 (1.9)	1,649 (21.8)	4,504 (20.5)	900 (26.7)	90 (14.6)	43 (23.1)	4,611 (44.0)	117,304 (23.3)	5,999 (17.7)	447 (17.2)	299 (20.9)	68 (12.7)	76 (16.3)	4,843 (26.6)	134 (6.7)
Asthma without COPD	1,560 (20.5)	700 (9.2)	3,374 (15.3)	529 (15.7)	119 (19.3)	37 (19.9)	1,148 (11.0)	81,396 (16.2)	3,346 (9.9)	479 (18.4)	221 (15.4)	52 (9.7)	89 (19.1)	953 (5.2)	80 (4.0)
Pregnant women	121 (1.6)	79 (1.0)	1,121 (5.1)	18 (0.5)	12 (2.0)	NR	NR	11,126 (2.2)	1,058 (3.1)	121 (4.7)	80 (5.6)	18 (3.4)	44 (9.4)	101 (0.6)	13 (0.6)
CKD	421 (5.5)	1,870 (24.7)	5,915 (26.9)	1,189 (35.3)	142 (23.1)	NR	3,937 (37.6)	162,943 (32.4)	7,718 (22.7)	661 (25.4)	344 (24.0)	131 (24.5)	100 (21.5)	2,658 (14.6)	178 (8.9)
ERSD	30 (0.4)	644 (8.5)	1,376 (6.2)	271 (8.0)	31 (5.0)	NR	1,513 (14.4)	53,074 (10.6)	3,053 (9.0)	359 (13.8)	96 (6.7)	39 (7.3)	28 (6.0)	NR	88 (4.4)
HIVAIDS	NR	55 (0.7)	190 (0.9)	21 (0.6)	NR	NR	235 (2.2)	6,893 (1.4)	456 (1.3)	67 (2.6)	12 (0.8)	NR	NR	47 (0.3)	13 (0.6)

Abbreviations: Clinical Practice Research Datalink (CPRD), Colorado University Anschuz Medical Campus Health Data Compass (CU-AMC HDC), Columbia University Irving Medical Center (CUIMC), Daegu Catholic University Medical Center (DCMC) Health Insurance Review & Assessment Service (HIRA), Hospital del Mar (HMAR), Integrated Primary Care Information (IPCI), IQVIA Disease Analyser Germany (DA Germany), IQVIA Longitudinal Patient Data France (LPD France), IQVIA Longitudinal Patient Data Italy (LPD Italy), Nanfang Hospital COVID-19 Research Database (NFHCRD) Oregon Health & Science University (OHSU) Information System for Research in Primary Care (SIDIAP), Information System for Research in Primary Care – Hospitalization Linked Data (SIDIAP-H), STAnford medicine Research data Repository (STARR-OMOP), Tufts MC Research Data Warehouse (TRDW), UW Medicine COVID Research Dataset (UWM-CRD) Department of Veterans Affairs (VA-OMOP), NR: Not Reported by Data Partner. COPD: Chronic obstructive pulmonary disease; CKD: Chronic kidney disease; ESRD: End-stage renal disease; HIV/AIDS: Human immunovirus deficiency/Acquired immunodeficiency syndrome.

Unraveling COVID-19: a large-scale characterization of 4.5 million COVID-19 cases using CHARYBDIS

Supplementary Table 6. Symptoms at index date and 30-day outcomes in persons diagnosed and/or hospitalized with a COVID-19 diagnosis or SARS-CoV-2 positive test in each cohort across the OHDSI COVID-19 Network*

	ASIA			UNITED STATES												EUROPE							
	HIRA	DCMC	NFHCRD	HealthVerity	Premier	OPTUM-EHR	OPTUM-SES	STARR-OMOP	TRDW	VA-OMOP	IQVIA OpenClaims	IQVIA Hospital CDM	CUIMC	UWM-CRD	OHSU	HM-Hospitales	SIDIAP	HMAR	IPCI	CPRD	IQVIA LPD France	IQVIA DA Germany	IQVIA LPD Italy
**Diagnosed**																							
**At index symptoms, %**																							
Dyspnea	NR	0.9	NR	1.8	17.1	7.6	21.5	3.3	14.6	4.7	7.4	6.3	11.8	4.5	4.7	NR	0.9	NR	0.9	0.9	1.2	0.6	NR
Cough	NR	NR	NR	6.4	16.4	9	22.5	6.6	32.2	7.3	8.5	12.4	7.1	6.5	13.3	NR	2.6	NR	1.8	0.7	6.2	1.7	1.3
Fever	NR	6.1	NR	4.7	16.7	6.8	21	5.2	23	4.6	6.7	10.3	5	5.3	15.6	NR	2.2	NR	1.4	0.2	4.9	1.5	NR
Malaise or fatigue	NR	NR	NR	1.9	4.5	2.2	5.1	1.8	1.3	1.5	2.1	2.9	1.3	1.6	2.4	NR	0.2	NR	0.4	0.2	2.5	0.3	0.2
Myalgia	NR	NR	NR	0.4	2.2	1.2	1.7	0.9	4.8	1	0.8	2.5	0.2	0.9	12	NR	0	NR	NR	0.2	0.3	0.1	NR
Anosmia OR Hyposmia OR Dysgeusia	NR	NR	NR	0.5	0.6	0.9	0.4	0.3	NR	0.5	0.8	1.9	0.3	NR	8.1	NR	0	NR	NR	NR	1.8	0.3	0.1
**30-day outcomes, %**																							
Hospitalization episodes	NR	8.2	59.3	3.4	54.3	17.7	43.9	15.1	19.8	16.6	18.6	15.5	28.7	17.5	5.3	NR	13.1	NR	1.3	NR	NR	NR	NR
Intensive services during hospitalization	NR	NR	1.2	0.2	12.2	2.1	5.9	1.3	6.5	2.9	1.6	3.3	0.6	2.7	0.5	NR	NR	NR	NR	NR	NR	NR	NR
Death	NR	NR	NR	NR	10.6	0.9	NR	0.3	2.6	3.6	NR	5.8	6.3	3.3	0.5	NR	4.3	NR	5.3	14.1	NR	0.1	NR
**Hospitalized**																							
**At index symptoms, %**																							
Dyspnea	8.8	NR	NR	11.8	16.6	23.8	34.7	7.3	6.4	13.9	14.4	14.7	32	23.2	8.9	0.3	2.4	0.8	NR	NR	NR	NR	NR
Cough	11.7	NR	NR	4.9	4.8	13.2	20.8	3.9	3.7	6	5	3.1	14	14.3	5.7	0.2	2.2	2.9	NR	NR	NR	NR	NR
Fever	5.2	NR	3.6	7.3	9.4	13.2	23.7	6.9	7.1	6.5	7	6.9	9.9	17.6	9.6	12.2	3.1	2.9	NR	NR	NR	NR	NR
Malaise or fatigue	NR	NR	NR	2.7	4.4	5.2	6.2	6.7	NR	2.4	2.3	1.9	3.3	5.6	3	0.7	0.3	1	NR	NR	NR	NR	NR
Myalgia	2.4	NR	NR	0.2	0.6	1.4	1.2	1.3	NR	0.6	0.2	0.1	NR	2.5	NR	NR	0	0.9	NR	NR	NR	NR	NR
Anosmia OR Hyposmia OR Dysgeusia	0.2	NR	NR	0.1	0.2	0.3	0.2	NR	NR	0.2	0.1	0	NR	NR	NR	NR	0	2.7	NR	NR	NR	NR	NR

**30-day outcomes, %**																									
Intensive services during hospitalization	1.7	NR	2		5.3	22.6	12	13.2	8.2	30.7	16.7	8.4	22.1	2.4	15.8	10		54.9	NR	8.2	NR	NR	NR	NR	NR
Death	2.5	NR	NR		NR	19	4.2	NR	0.9	9.5	14.5	NR	14.6	18.8	13.6	6.7		14.1	12.2	5.9	NR	NR	NR	NR	NR

*Proportions presented among diagnosed or hospitalized patients with a COVID-19 diagnosis or SARS-CoV-2 positive test by database (column percentage); - data not available; < values were below the minimum cell count required (5 individuals); no prior observation time was required.

**Prevalent conditions at index date.

Abbreviations: Clinical Practice Research Datalink (CPRD), Colorado University Anschuz Medical Campus Health Data Compass (CU-AMC HDC), Columbia University Irving Medical Center (CUIMC), Daegu Catholic University Medical Center (DCMC) Health Insurance Review & Assessment Service (HIRA), Hospital del Mar (HMAR), Integrated Primary Care Information (IPCI), IQVIA Disease Analyser Germany (DA Germany), IQVIA Longitudinal Patient Data France (LPD France), IQVIA Longitudinal Patient Data Italy (LPD Italy), Nanfang Hospital COVID-19 Research Database (NFHCRD) Oregon Health & Science University (OHSU) Information System for Research in Primary Care (SIDIAP), Information System for Research in Primary Care – Hospitalization Linked Data (SIDIAP-H), STAnford medicine Research data Repository (STARR-OMOP), Tufts MC Research Data Warehouse (TRDW), UW Medicine COVID Research Dataset (UWM-CRD) Department of Veterans Affairs (VA-OMOP), NR: Not Reported by Data Partner. COPD: Chronic obstructive pulmonary disease; CKD: Chronic kidney disease; ESRD: End-stage renal disease; HIV/AIDS: Human immunovirus deficiency/Acquired immunodeficiency syndrome.
