# Supplementary material for: Unraveling COVID-19: a large-scale characterization of 4.5 million COVID-19 cases using CHARYBDIS
Source: Res Sq. 2021 Mar 1:rs.3.rs-279400. Preprint. [Version 1] doi: 10.21203/rs.3.rs-279400/v1 (PMC7941629; doi:10.21203/rs.3.rs-279400/v1)
Supplement: Supplement [file 68432cb4dd06b331efec35a0.docx]

# Table 1. Characteristics of persons with a COVID-19 diagnosis or SARS-CoV-2 positive test across the OHDSI COVID-19 Network*

|  | **ASIA** | |  | **UNITED STATES** | | | | | | | | | | | | |  | **EUROPE** | | | | | |
| --- | --- | --- | --- | --- | --- | --- | --- | --- | --- | --- | --- | --- | --- | --- | --- | --- | --- | --- | --- | --- | --- | --- | --- |
|  | **DCMC** | **NFHCRD** |  | **HealthVerity** | **Premier** | **OPTUM-EHR** | **OPTUM-SES** | **STARR-OMOP** | **TRDW** | **VA-OMOP** | **IQVIA-OpenClaims** | **IQVIA Hospital CDM** | **CUIMC** | **CU-AMC-HDC** | **UWM-CRD** | **OHSU** |  | **SIDIAP** | **IPCI** | **CPRD** | **IQVIA LPD France** | **IQVIA DA Germany** | **IQVIA LPD Italy** |
| COVID-19 Cases (N) | 559 | 403 |  | 587,683 | 66,132 | 160,613 | 7,863 | 4,788 | 1,250 | 57,937 | 2,785,812 | 153,477 | 10,437 | 9,481 | 3,245 | 11,187 |  | 124,305 | 3,306 | 3,864 | 23,592 | 11,500 | 4,816 |
| Persons Tested | NR | 397 |  | 3,898,593 | 219,230 | 1,025,584 | 41,673 | 56,881 | 6,950 | 521,814 | 6,520,151 | 719,596 | 22,094 | 120,661 | 83,921 | 109,434 |  | 173,957 | NR | 5,551 | NR | NR | NR |
| Tested Positive, n (%)* | NR | 392 (97.3) |  | 425,610 (72.4) | NR | 73,113 (45.5) | NR | 1,880 (39.3) | 1,035 (82.8) | 32,847 (56.7) | NR | NR | 6,959 (66.7) | NR | 3,140 (96.8) | 8,764 (78.3) |  | 39,047 (31.4) | NR | 2,098 (54.3) | NR | NR | NR |
|  | | | | | | | | | | | | | | | | | | | | | | | |
| Full 30-day follow up | 162 (29.0) | 276 (68.5) |  | 67,071 (11.4) | 3,902 (5.9) | 84,073 (52.3) | 1,269 (16.1) | 2,703 (56.5) | 641 (51.3) | 44,661 (77.1) | 1,882,950 (67.6) | 21,145 (13.8) | 2,008 (19.2) | 8,755 (92.3) | 1,199 (36.9) | 3,760 (33.6) |  | 81,914 (65.9) | 2,601 (78.7) | 2,723 (70.5) | 9,819 (41.6) | 5,588 (48.6) | 3,570 (74.1) |
| < 30-day follow up | 397 (71.0) | 127 (31.5) |  | 520,612 (88.6) | 62,230 (94.1) | 76,540 (47.7) | 6,594 (83.9) | 2,085 (43.5) | 609 (48.7) | 13,272 (22.9) | 902,862 (32.4) | 132,332 (86.2) | 8,429 (80.8) | 706 (7.4) | 2,046 (63.1) | 7,427 (66.4) |  | 42,391 (34.1) | 705 (21.3) | 1,141 (29.5) | 13,773 (58.4) | 5,912 (51.4) | 1,246 (25.9) |
| **Comorbidities, n (%)**** | | | | | | | | | | | | | | | | | | | | | | | |
| Type 2 Diabetes Mellitus | 108 (19.3) | 9 (2.2) |  | 20,922 (3.6) | 10,783 (16.3) | 26,897 (16.7) | 2,673 (34.0) | 555 (11.6) | 179 (14.3) | 19,083 (32.9) | 724,991 (26.0) | 35,576 (23.2) | 1,977 (18.9) | 1,396 (14.7) | 391 (12.0) | 603 (5.4) |  | 9,941 (8.0) | 500 (15.1) | 545 (14.1) | 1,318 (5.6) | 1,089 (9.5) | 452 (9.4) |
| Hypertension | 154 (27.5) | 19 (4.7) |  | 34,090 (5.8) | 19,008 (28.7) | 54,678 (34.0) | 4,393 (55.9) | 1,319 (27.5) | 307 (24.6) | 34,357 (59.3) | 1,260,816 (45.3) | 60,495 (39.4) | 3,771 (36.1) | 2,708 (28.6) | 735 (22.7) | 1,065 (9.5) |  | 21,337 (17.2) | 688 (20.8) | 779 (20.2) | 3,522 (14.9) | 2,611 (22.7) | 1,659 (34.4) |
| Heart disease | 106 (19.0) | 7 (1.7) |  | 19,016 (3.2) | 11,533 (17.4) | 39,510 (24.6) | 3,726 (47.4) | 977 (20.4) | 245 (19.6) | 24,699 (42.6) | 936,271 (33.6) | 33,846 (22.1) | 3,236 (31.0) | 1,871 (19.7) | 440 (13.6) | 778 (7.0) |  | 17,759 (14.3) | 470 (14.2) | 722 (18.7) | 1,213 (5.1) | 2,007 (17.5) | 1,013 (21.0) |
| History of cancer | 32 (5.7) | NR |  | 6,107 (1.0) | 3,157 (4.8) | 18,536 (11.5) | 1,491 (19.0) | 887 (18.5) | 106 (8.5) | 10,792 (18.6) | 317,479 (11.4) | 11,237 (7.3) | 1,480 (14.2) | 843 (8.9) | 184 (5.7) | 469 (4.2) |  | 8,872 (7.1) | 262 (7.9) | 296 (7.7) | 674 (2.9) | 661 (5.7) | 547 (11.4) |
| Hepatitis C | NR | NR |  | 740 (0.1) | 410 (0.6) | 1,395 (0.9) | 112 (1.4) | 61 (1.3) | 35 (2.8) | 3,075 (5.3) | 40,101 (1.4) | 1,966 (1.3) | 144 (1.4) | 90 (0.9) | 54 (1.7) | 88 (0.8) |  | 648 (0.5) | NR | NR | 40 (0.2) | 31 (0.3) | 53 (1.1) |
| Obesity | 29 (5.2) | NR |  | 15,072 (2.6) | 7,298 (11.0) | 71,076 (44.3) | 2,468 (31.4) | 1,246 (26.0) | 325 (26.0) | 25,128 (43.4) | 740,430 (26.6) | 28,757 (18.7) | 3,729 (35.7) | 3,136 (33.1) | 233 (7.2) | 945 (8.4) |  | 36,557 (29.4) | 629 (19.0) | 1,428 (37.0) | 2,287 (9.7) | 1,345 (11.7) | 674 (14.0) |
| Dementia | 6 (1.1) | NR |  | 4,255 (0.7) | 3,697 (5.6) | 5,360 (3.3) | 851 (10.8) | 38 (0.8) | 29 (2.3) | 4,019 (6.9) | 219,062 (7.9) | 7,776 (5.1) | 483 (4.6) | 235 (2.5) | 116 (3.6) | 97 (0.9) |  | 6,013 (4.8) | 64 (1.9) | 327 (8.5) | 55 (0.2) | 339 (2.9) | 81 (1.7) |
| Autoimmune condition | 49 (8.8) | NR |  | 7,291 (1.2) | 1,678 (2.5) | 13,396 (8.3) | 1,464 (18.6) | 418 (8.7) | 133 (10.6) | 10,103 (17.4) | 433,259 (15.6) | 8,965 (5.8) | 1,388 (13.3) | 720 (7.6) | 140 (4.3) | 409 (3.7) |  | 8,260 (6.6) | 476 (14.4) | 394 (10.2) | 1,467 (6.2) | 1,183 (10.3) | 636 (13.2) |
| Chronic obstructive pulmonary disease (COPD) without asthma | NR | NR |  | 8,160 (1.4) | 3,335 (5.0) | 12,067 (7.5) | 1,449 (18.4) | 231 (4.8) | 89 (7.1) | 12,665 (21.9) | 297,269 (10.7) | 12,008 (7.8) | 809 (7.8) | 733 (7.7) | 112 (3.5) | 249 (2.2) |  | 15,819 (12.7) | 213 (6.4) | 294 (7.6) | 696 (3.0) | 868 (7.5) | 350 (7.3) |
| Asthma without COPD | 17 (3.0) | NR |  | 10,458 (1.8) | 3,972 (6.0) | 21,076 (13.1) | 1,125 (14.3) | 521 (10.9) | 112 (9.0) | 6,278 (10.8) | 438,892 (15.8) | 12,936 (8.4) | 1,376 (13.2) | 1,100 (11.6) | 176 (5.4) | 567 (5.1) |  | 7,567 (6.1) | 322 (9.7) | 494 (12.8) | 2,327 (9.9) | 1,097 (9.5) | 420 (8.7) |
| Pregnant women | NR | NR |  | 3,543 (0.6) | 1,192 (1.8) | 3,917 (2.4) | 109 (1.4) | 52 (1.1) | 27 (2.2) | 86 (0.1) | 41,329 (1.5) | 2,944 (1.9) | 382 (3.7) | 212 (2.2) | 32 (1.0) | 156 (1.4) |  | 689 (0.6) | 32 (1.0) | 11 (0.3) | 212 (0.9) | 39 (0.3) | 63 (1.3) |
| Chronic kidney disease broad | 156 (27.9) | NR |  | 7,535 (1.3) | 5,711 (8.6) | 17,531 (10.9) | 1,829 (23.3) | 398 (8.3) | NR | 10,239 (17.7) | 364,857 (13.1) | 16,250 (10.6) | 1,181 (11.3) | 723 (7.6) | 213 (6.6) | 277 (2.5) |  | 8,144 (6.6) | 197 (6.0) | 478 (12.4) | 194 (0.8) | 562 (4.9) | 192 (4.0) |
| End stage renal disease | 155 (27.7) | NR |  | 1,683 (0.3) | 1,062 (1.6) | 3,008 (1.9) | 359 (4.6) | 122 (2.5) | NR | 3,273 (5.6) | 96,555 (3.5) | 5,155 (3.4) | 600 (5.7) | 166 (1.8) | 51 (1.6) | 52 (0.5) |  | 8 (0.0) | NR | 17 (0.4) | NR | 27 (0.2) | NR |
| Human immunodeficiency virus infection | NR | NR |  | 829 (0.1) | 357 (0.5) | 763 (0.5) | 67 (0.9) | 20 (0.4) | NR | 817 (1.4) | 24,808 (0.9) | 1,309 (0.9) | 163 (1.6) | 56 (0.6) | 45 (1.4) | 43 (0.4) |  | 290 (0.2) | NR | NR | 83 (0.4) | 18 (0.2) | 19 (0.4) |

*Proportions presented among diagnosed patients with a COVID-19 diagnosis or SARS-CoV-2 positive test by database (column percentage); since SIDIAP_H includes a subset of SIDIAP, results were not included in this table; - data not available or below the minimum cell count required (5 individuals); no prior observation time was required.

**Prevalent conditions at index date.

Abbreviations:

CU-AMC-HDC: U of Colorado Anschuz Medical Campus Health Data Compass; CUIMC: Columbia University Irving Medical Center; IQVIAHospitalCDM: IQVIA Hospital Charge Data Master; OHSU: Oregon Health and Science University; OPTUM-EHR: Optum© de-identified Electronic Health Record Dataset; OPTUM-SES: Optum® De-Identified Clinformatics® Data Mart Database – Socio-Economic Status (SES); STARR-OMOP: Stanford Medicine Research Data Repository; TRDW: Tufts Research Data Warehouse; UWM-CRD: UW Medicine COVID Research Dataset; VA-OMOP: Department of Veterans Affairs; NR: Not reported by data partner

# Table 2. Characteristics of persons hospitalized with a COVID-19 diagnosis or SARS-CoV-2 positive test across the OHDSI COVID-19 Network*

|  | **ASIA** | |  | **UNITED STATES** | | | | | | | | | | | | |  | **EUROPE** | | |
| --- | --- | --- | --- | --- | --- | --- | --- | --- | --- | --- | --- | --- | --- | --- | --- | --- | --- | --- | --- | --- |
|  | **HIRA** | **NFHCRD** |  | **HealthVerity** | **Premier** | **OPTUM-EHR** | **OPTUM-SES** | **STARR-OMOP** | **TRDW** | **VA-OMOP** | **IQVIA OpenClaims** | **IQVIA Hospital CDM** | **CUIMC** | **CU-AMC-HDC** | **UWM-CRD** | **OHSU** |  | **HM Hospitals** | **SIDIAP** | **HMAR** |
| **COVID-19 Cases (N)** | 7,599 | 304 |  | 22,887 | 36,019 | 29,061 | 4,336 | 744 | 326 | 10,951 | 533,997 | 57,062 | 3,439 | 1,874 | 733 | 627 |  | 2,544 | 18,369 | 2,686 |
| **Hospitalized with positive test, n (%)** | NR | 125 (41.1) |  | 13,262 (57.9) | NR | 13,817 (47.5) | NR | 128 (17.2) | 232 (71.2) | 8,623 (78.7) | NR | NR | 3,075 (89.4) | NR | 676 (92.2) | 344 (54.9) |  | NR | 13,685 (74.5) | 773 (28.8) |
|  | | | | | | | | | | | | | | | | | | | | |
| Full 30-day follow up | 7,359 (96.8) | 284 (93.4) |  | 10,333 (45.1) | 2,361 (6.6) | 18,555 (63.8) | 851 (19.6) | 657 (88.3) | NR | 8,548 (78.1) | 412,537 (77.3) | 11,876 (20.8) | 943 (27.4) | 1,810 (96.6) | 400 (54.6) | 484 (77.2) |  | 109 (4.3) | 12,290 (66.9) | 1,254 (46.7) |
| < 30-day follow up | 240 (3.2) | 20 (6.6) |  | 12,554 (54.9) | 33,658 (93.4) | 10,506 (36.2) | 3,485 (80.4) | 87 (11.7) | NR | 2,400 (21.9) | 121,460 (22.7) | 45,186 (79.2) | 2,496 (72.6) | 64 (3.4) | 333 (45.4) | 143 (22.8) |  | 2,435 (95.7) | 6,079 (33.1) | 1,432 (53.3) |
|  | | | | | | | | | | | | | | | | | | | | |
| Type 2 Diabetes Mellitus | 1,760 (23.2) | NR |  | 3,880 (17.0) | 8,899 (24.7) | 9,531 (32.8) | 1,844 (42.5) | 157 (21.1) | 83 (25.5) | 5,839 (53.3) | 254,505 (47.7) | 16,480 (28.9) | 1,120 (32.6) | 677 (36.1) | 226 (30.8) | 177 (28.2) |  | 428 (16.8) | 3,295 (17.9) | 294 (10.9) |
| Hypertension | 1,943 (25.6) | NR |  | 6,410 (28.0) | 15,216 (42.2) | 16,427 (56.5) | 2,977 (68.7) | 389 (52.3) | 123 (37.7) | 9,087 (83.0) | 390,171 (73.1) | 26,262 (46.0) | 1,770 (51.5) | 1,073 (57.3) | 398 (54.3) | 283 (45.1) |  | 1,139 (44.8) | 5,645 (30.7) | 653 (24.3) |
| Heart disease | 1,271 (16.7) | NR |  | 5,178 (22.6) | 10,384 (28.8) | 13,274 (45.7) | 2,634 (60.7) | 297 (39.9) | 109 (33.4) | 7,421 (67.8) | 319,842 (59.9) | 16,165 (28.3) | 1,534 (44.6) | 802 (42.8) | 286 (39.0) | 258 (41.1) |  | 606 (23.8) | 5,148 (28.0) | 362 (13.5) |
| History of cancer | 410 (5.4) | NR |  | 1,132 (4.9) | 2,811 (7.8) | 4,939 (17.0) | 1,065 (24.6) | 277 (37.2) | 47 (14.4) | 3,401 (31.1) | 106,805 (20.0) | 5,524 (9.7) | 588 (17.1) | 300 (16.0) | 90 (12.3) | 154 (24.6) |  | 286 (11.2) | 2,616 (14.2) | 179 (6.7) |
| Hepatitis C | 61 (0.8) | NR |  | 134 (0.6) | 394 (1.1) | 469 (1.6) | 77 (1.8) | 17 (2.3) | 13 (4.0) | 1,037 (9.5) | 14,408 (2.7) | 1,050 (1.8) | 81 (2.4) | 37 (2.0) | 25 (3.4) | 37 (5.9) |  | 15 (0.6) | 135 (0.7) | 38 (1.4) |
| Obesity | 16 (0.2) | NR |  | 2,238 (9.8) | 6,678 (18.5) | 15,497 (53.3) | 1,626 (37.5) | 312 (41.9) | 126 (38.7) | 5,677 (51.8) | 191,071 (35.8) | 10,735 (18.8) | 1,651 (48.0) | 988 (52.7) | 138 (18.8) | 167 (26.6) |  | 149 (5.9) | 8,428 (45.9) | 259 (9.6) |
| Dementia | 436 (5.7) | NR |  | 1,815 (7.9) | 3,428 (9.5) | 2,376 (8.2) | 637 (14.7) | 17 (2.3) | 17 (5.2) | 2,087 (19.1) | 81,638 (15.3) | 4,044 (7.1) | 373 (10.8) | 140 (7.5) | 95 (13.0) | 25 (4.0) |  | 108 (4.2) | 1,102 (6.0) | 75 (2.8) |
| Autoimmune condition | 813 (10.7) | NR |  | 1,215 (5.3) | 1,432 (4.0) | 3,320 (11.4) | 931 (21.5) | 89 (12.0) | 54 (16.6) | 3,156 (28.8) | 136,735 (25.6) | 4,205 (7.4) | 570 (16.6) | 226 (12.1) | 67 (9.1) | 83 (13.2) |  | 121 (4.8) | 1,706 (9.3) | 93 (3.5) |
| Chronic obstructive pulmonary disease (COPD) without asthma | 145 (1.9) | NR |  | 2,213 (9.7) | 3,016 (8.4) | 5,176 (17.8) | 1,066 (24.6) | 102 (13.7) | 52 (16.0) | 4,641 (42.4) | 118,421 (22.2) | 7,071 (12.4) | 469 (13.6) | 333 (17.8) | 77 (10.5) | 90 (14.4) |  | 173 (6.8) | 4,848 (26.4) | 138 (5.1) |
| Asthma without COPD | 1,560 (20.5) | NR |  | 1,004 (4.4) | 2,677 (7.4) | 3,746 (12.9) | 628 (14.5) | 127 (17.1) | 39 (12.0) | 1,153 (10.5) | 82,087 (15.4) | 3,825 (6.7) | 498 (14.5) | 245 (13.1) | 58 (7.9) | 101 (16.1) |  | 112 (4.4) | 957 (5.2) | 99 (3.7) |
| Pregnant women | 121 (1.6) | NR |  | 341 (1.5) | 682 (1.9) | 1,550 (5.3) | 30 (0.7) | 18 (2.4) | 13 (4.0) | NR | 12,748 (2.4) | 2,029 (3.6) | 158 (4.6) | 111 (5.9) | 22 (3.0) | 73 (11.6) |  | 7 (0.3) | 108 (0.6) | 20 (0.7) |
| Chronic kidney disease broad | 421 (5.5) | NR |  | 2,622 (11.5) | 5,339 (14.8) | 6,596 (22.7) | 1,357 (31.3) | 162 (21.8) | NR | 3,958 (36.1) | 164,710 (30.8) | 8,827 (15.5) | 691 (20.1) | 375 (20.0) | 152 (20.7) | 112 (17.9) |  | 157 (6.2) | 2,658 (14.5) | 186 (6.9) |
| End stage renal disease | 30 (0.4) | NR |  | 826 (3.6) | 948 (2.6) | 1,506 (5.2) | 296 (6.8) | 31 (4.2) | NR | 1,520 (13.9) | 53,747 (10.1) | 3,333 (5.8) | 371 (10.8) | 101 (5.4) | 43 (5.9) | 29 (4.6) |  | 7 (0.3) | NR | 91 (3.4) |
| Human immuno-deficiency virus infection | NR | NR |  | 96 (0.4) | 275 (0.8) | 222 (0.8) | 33 (0.8) | NR | NR | 239 (2.2) | 7,009 (1.3) | 516 (0.9) | 73 (2.1) | 14 (0.7) | 11 (1.5) | 11 (1.8) |  | NR | 47 (0.3) | 14 (0.5) |

*Proportions presented among diagnosed patients with a COVID-19 diagnosis or SARS-CoV-2 positive test by database (column percentage); - data not available or below the minimum cell count required (5 individuals); no prior observation time was required.

**Prevalent conditions at index date.

Abbreviations: CU-AMC-HDC: U of Colorado Anschuz Medical Campus Health Data Compass; CUIMC: Columbia University Irving Medical Center; IQVIAHospitalCDM: IQVIA Hospital Charge Data Master; OHSU: Oregon Health and Science University; OPTUM-EHR: Optum© de-identified Electronic Health Record Dataset; OPTUM-SES: Optum® De-Identified Clinformatics® Data Mart Database – Socio-Economic Status (SES); STARR-OMOP: Stanford Medicine Research Data Repository; TRDW: Tufts Research Data Warehouse; UWM-CRD: UW Medicine COVID Research Dataset; VA-OMOP: Department of Veterans Affairs; HM-Hospitals: HM-Hospitals Madrid; SIDIAP: Information System for Research in Primary Care; HMAR: Hospital del Mar; NR: Not reported by data partner
